# Unveiling previously undescribed circadian clock regulators and action mechanisms via TurboID-based profiling of the LWD1 interactome in *Arabidopsis thaliana*

**DOI:** 10.1073/pnas.2618203123

**Published:** 2026-08-05

**Authors:** Chun-Kai Huang, Chuan-Chih Hsu, Huang-Lung Tsai, Wen-Dar Lin, Jing-Fen Wu, Shu-Hsing Wu

**Affiliations:** ^a^ Institute of Plant and Microbial Biology, Academia Sinica, Taipei 115201, Taiwan; ^b^ Institute of Molecular and Cellular Biology, National Taiwan University, Taipei 10617, Taiwan

**Keywords:** circadian clock, proximity labeling, epigenetic regulators, transcriptional repressors, TurboID

## Abstract

The synchronization of the internal circadian clock with the external 24-h diel cycle is critical for plant growth and fitness. LIGHT-REGULATED WD proteins (LWDs) are essential for sustaining a precise and robust circadian clock in *Arabidopsis* [Y. Wang *et al.*, *Plant Cell* **23**, 486–498 (2011); J. F. Wu *et al.*, *Nat Commun.* **7**, 13181 (2016)]. However, the action mechanisms by which LWDs orchestrate the expression of clock genes remain largely elusive. We systematically identified 219 LWD1-interacting protein partners at three Zeitgeber times (ZTs) using a TurboID-mediated proximity labeling approach. Selected interacting proteins were experimentally verified as new regulators of the *Arabidopsis* circadian clock. Our data also support that LWD1-PSEUDO RESPONSE REGULATORS (PRRs)-TOPLESS (TPL) complex represses the morning gene *CIRCADIAN CLOCK ASSOCIATED* (*CCA1*), shaping the expression trough of *CCA1* in the late afternoon/early evening under diel cycles. Our results revealed a new corepressor role of LWD1, working in conjunction with epigenetic regulators and transcription factors to underpin the precise operation of the circadian clock.

## Significance

Elucidating the mechanisms that sustain circadian precision is crucial for understanding how plants align their internal rhythms with external environmental cycles. This study provides mechanistic insights into the action roles of LWD1 by constructing its time-resolved protein interaction map, thereby uncovering new clock regulators that interact with LWD1. Importantly, a new role of LWD1 in recruiting transcriptional repressors to attenuate the expression of *CIRCADIAN CLOCK ASSOCIATED 1* (*CCA1*) was experimentally validated, complementing its previously recognized coactivator function before dawn. Beyond refining our understanding of circadian architecture, this mechanistic knowledge lays the groundwork for advancing current models of clock function and improving our understanding of how plants synchronize with their environment.

The circadian clock, an intrinsic timekeeping mechanism with a cycle of approximately 24 h, is pivotal for the prosperous growth and development of living organisms on Earth. For sessile plants, the built-in circadian clock orchestrates the expression of thousands of genes in response to daily light and temperature changes, allowing plants to adapt and respond efficiently to their surroundings (1–4). Functional operation of the circadian clock ensures the robustness and precision of critical physiological processes such as photosynthesis efficiency, stress responses, and flowering time control, consequently impacting growth and reproduction (1, 5–8). A deeper understanding of the establishment of the circadian clock, as well as its role in interpreting and synchronizing with the external environment, has profound implications for agriculture.

In the current model of the *Arabidopsis* circadian clock, the daily oscillation is mainly driven by the repressilator machinery and assisted by several activator/coactivator complexes to maintain the robustness of the circadian rhythm (9–11). *Arabidopsis* mutant defective in *LIGHT-REGULATED WD protein 1* (*LWD1*) and *LWD2* is early-flowering and has a significantly shortened period length (~18 h) and circadian amplitudes, highlighting the essential role of LWDs in the *Arabidopsis* circadian clock (12, 13).

LWDs do not harbor DNA-binding domains but possess multiple WD-repeats that can be protein–protein interaction scaffolds for the assembly of protein complexes (14). Indeed, LWD1 can partner with TEOSINATE BRANCHED 1-CYCLOIDEA-PCF20 (TCP20) or TCP22, to efficiently activate the expression of the morning gene *CIRCADIAN CLOCK ASSOCIATED* (*CCA1*) in anticipation of dawn (9). In the absence of LWD proteins, TCP20 and TCP22 alone are insufficient to activate *CCA1* expression, suggesting LWDs are essential coactivators (9). In addition to regulating *CCA1* activation, LWD1 directly associates with the promoters of multiple core clock oscillator genes and is required for keeping the expression of these genes at their full scales and at the right times during a 24-h day (12). The multiple entry points of LWD1 in the *Arabidopsis* circadian clock and its scaffold protein nature prompted us to investigate if LWD1 has additional protein partners and how they together facilitate the establishment and operation of the *Arabidopsis* circadian clock.

## Results

### LWD1-TurboID Fusion Protein Is Biologically Functional.

**Fig. 1.**
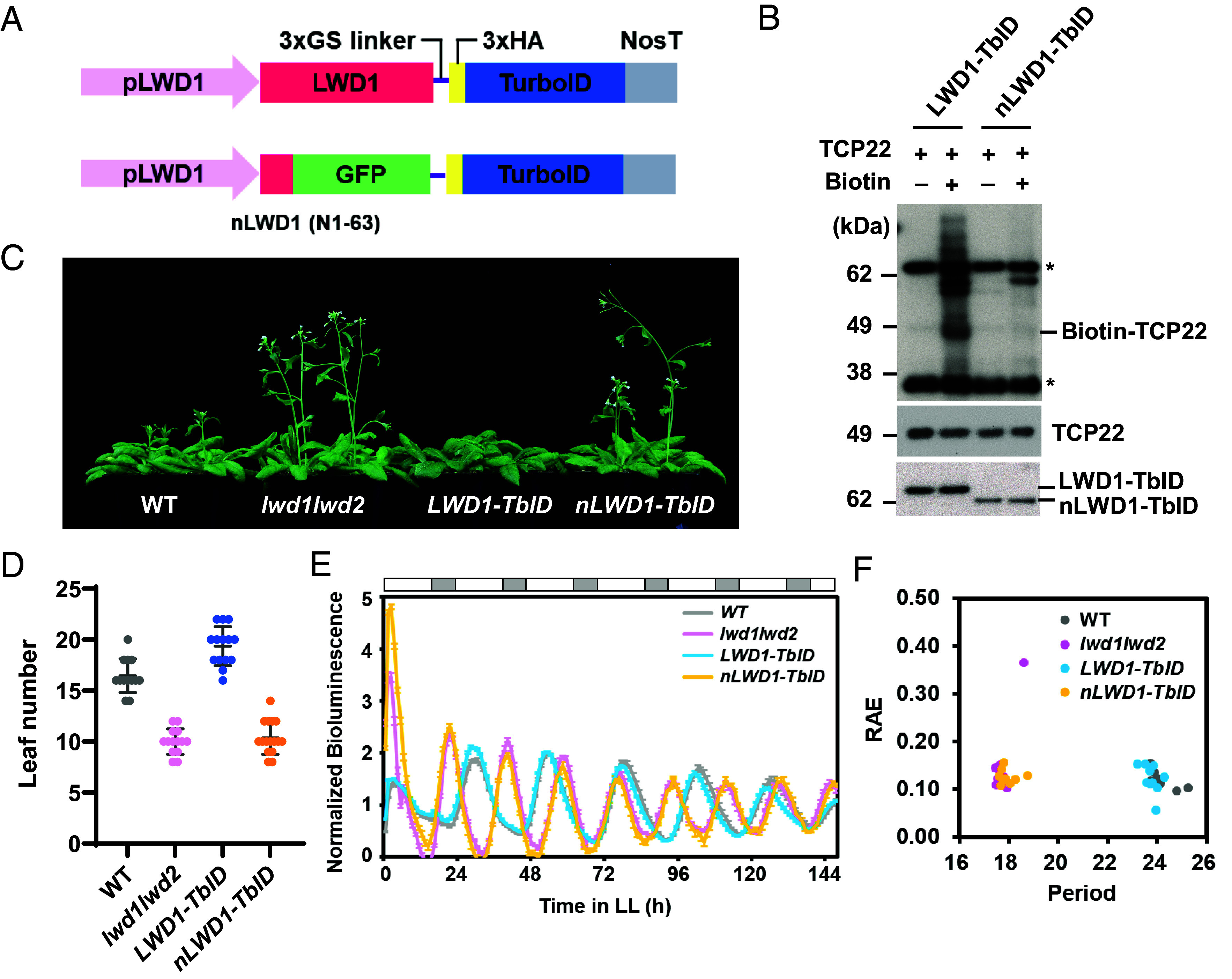
LWD1-TbID interacts with TCP22 and functionally complements *lwd1lwd2* phenotypes. (*A*) Diagram of two constructs used for LWD1-TurboID (LWD1-TbID) proximity labeling and the control, nLWD1-GFP-TurboID (nLWD1-TbID). LWD1 or N-terminal 63 amino acid of LWD1 (nLWD1_1-63_, hereafter as nLWD 1) was fused with 3 repeats of GGGGS linker and a 3xHA tag at the N terminus of TurboID under the control of the *LWD1* promoter (*pLWD1*) and nopaline synthase terminator (*NosT*). Green fluorescent protein (GFP) was used to fill in the protein size in nLWD1-TbID. (*B*) Transient expression of LWD1-TbID efficiently biotinylates TCP22 in *Arabidopsis* protoplasts. *LWD1-TbID* or *nLWD1-TbID* were coexpressed with *TCP22-FLAG* in *Arabidopsis* protoplasts with or without 50 μM biotin at ZT18. Proteins extracted at ZT4 in the following day were used for the detection of LWD1-TbID, nLWD1-TbID, and TCP22 with anti-HA or anti-FLAG antisera. Biotinylated proteins were detected by using streptavidin-horseradish peroxidase (Strep-HRP), and the biotinylated TCP22 (Biotin-TCP22) was marked. Asterisks indicated nonspecific bands. (*C*) Flowering phenotype of the wild type (WT), *lwd1lwd2*, *LWD1-TboID in lwd1lwd2* (*LWD1-TbID*), and *nLWD1-TbID in lwd1lwd2* (*nLWD1-TbID*) grown in long-day condition (16-h light/ 8-h dark) with ~80 μmole m^−2^s^−1^ white light illumination. (*D*) Flowering time represented by total rosette leaf numbers for WT, *lwd1lwd2*, *LWD1-TbID*, and *nLWD1-TbID*. Data are means with SD of ≥9 plants. Three biological replicates showed similar results. (*E*) Data are means with SE of ≥ 9 seedlings. White and gray bars denote the light and subjective darkness, respectively. The normalized promoter activities of morning gene *CCA1* were measured by bioluminescence assays for *pCCA1:LUC2* in WT, *lwd1lwd2*, *LWD1-TbID*, and *nLWD1-TbID* lines as described in *Materials and Methods*. (*F*) Period length and relative amplitude error (RAE) of WT, *lwd1lwd2, LWD1-TbID,* and *nLWD1-TbID* seedlings were calculated by the FFT-NLLS analysis from CT48 to CT120. Three independent experiments were performed with similar results.

We used the TurboID proximity labeling approach (15) to systematically identify proteins interacting with LWD1 *in planta*. LWD1-TurboID fusion construct was created by fusing LWD1 with biotin ligase, separated by a flexible 3xGS linker to reduce possible spatial constraints that can compromise proximity labeling efficiency (16). The construct was termed *pLWD1:LWD1-GS-HA-TurboID* and hereinafter referred to as *LWD1-TbID* (Fig. 1*A*). For a control, N-terminal 63 aa of LWD1 (nLWD1), containing only the predicted nuclear localization signal (NLS) but not the WD repeats (*SI Appendix*, Fig. S1 *A* and *B*), was fused with green fluorescence protein (GFP) to fill up the protein size, and TurboID to generate *pLWD1:nLWD1-GFP-GS-HA-TurboID* (*nLWD1-TbID*) (Fig. 1*A*). Like LWD1-GFP, nLWD1-TbID proteins reside in the nucleus as predicted (*SI Appendix*, Fig. S1 *C* and *D*). Both LWD1-TbID and nLWD1-TbID expressed with predicted sizes (*SI Appendix*, Fig. S1*E*).

The known LWD1-interacting protein TCP22 (9) can only be biotinylated by LWD1-TbID, but not nLWD1-TbID, in the presence of biotin in transient assays (Fig. 1*B*). This indicates that LWD1-TbID can effectively label its proximal protein partners and WD repeats are required for LWD1 to interact with TCP22, and likely other LWD1-interacting proteins. Also, only LWD1-TbID, but not nLWD1-TbID, can fully complement the early flowering and short period length phenotypes of the *lwd1lwd2* mutant (Fig. 1 *C*–*F* and *SI Appendix*, Fig. S1*F*). Therefore, transgenic *lwd1lwd2* expressing biologically functional LWD1-TbID and the negative control nLWD1-TbID were used as materials to profile the LWD1 interactome.

### LWD1 Interacts With Functionally Multifaceted Proteins.

**Fig. 2.**
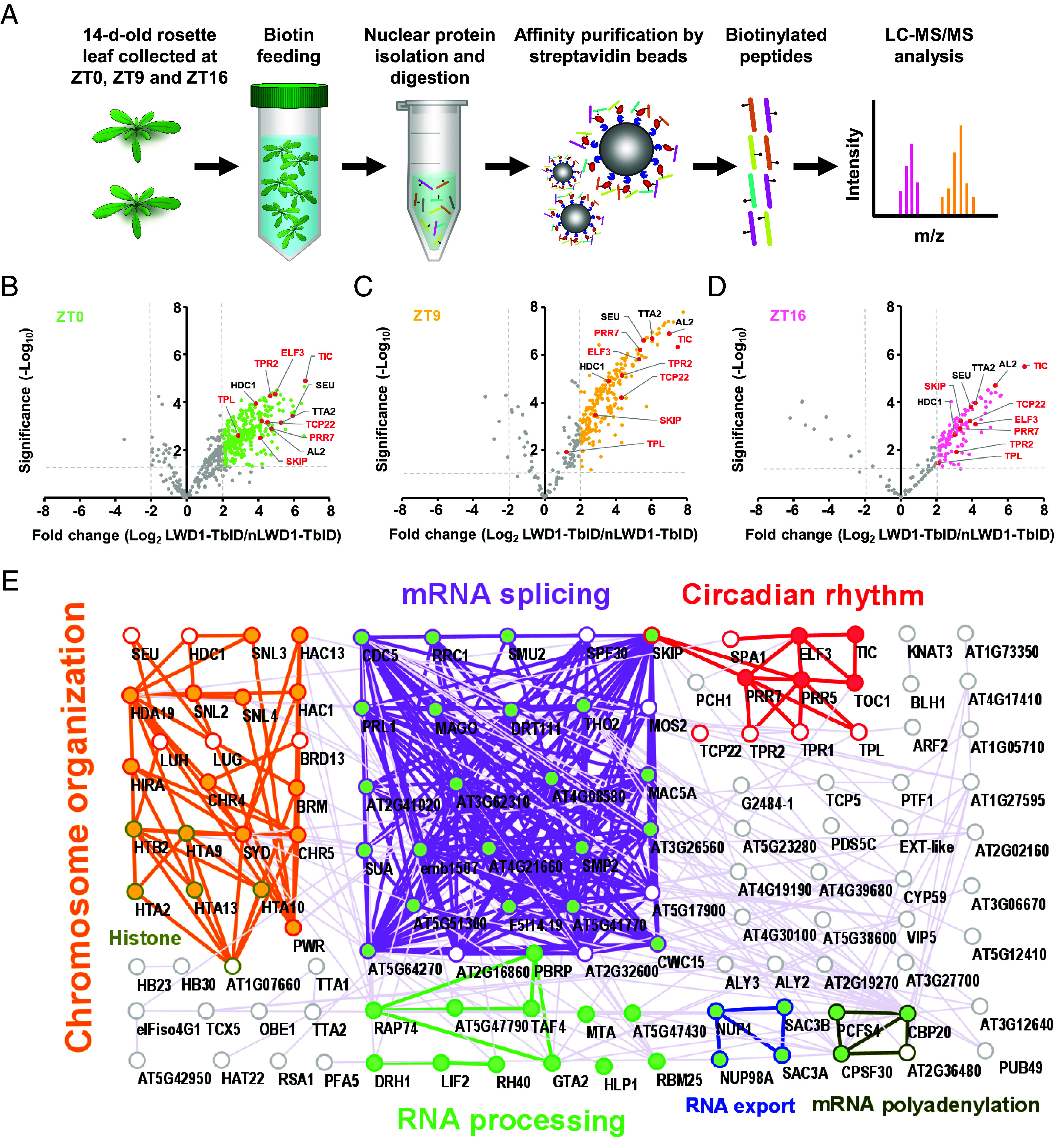
Proximity labeling reveals potential LWD1-interacting proteins. (*A*) Schematic diagram showing experimental procedures from sample preparation to protein identification by liquid chromatography coupled to tandem mass spectrometry (LC–MS/MS). The detailed procedures were described in *Materials and Methods*. (*B*–*D*) Volcano plots showing the significant enrichment of biotinylated peptides identified in LWD1-TbID compared to nLWD1-TbID at ZT0 (*B*), ZT9 (*C*), and ZT16 (*D*). The green dots in ZT0, orange dots in ZT9 and pink dots in ZT16 represent peptides with log_2_(LWD1-TbID/nLWD1-TbID) fold changes ≥2 and significance level of *P*-values ≤ 0.05. Names for proteins detected in all 3 ZTs and annotated as known and new clock proteins validated in this study are marked in red and black, respectively. (*E*) Biotinylated proteins identified were used to interrogate the protein interaction network using String database, followed by gene ontology (GO) categorization. Protein–protein interaction edges and circle outlines are color-coded according to their enrichment in designated GO terms shown. Filled circles denote those in higher-order GO categories.

LWD1 has different affinities toward the promoters of *CCA1*, *PSEUDO RESPONSE REGULATORS (PRRs),* and *TIMING OF CAB EXPRESSION1 (TOC1)* at different times of the day (9, 12). This implies that LWD1 may partner with different transcriptional regulators throughout the day to modulate the expression of clock genes. The pursuit of the LWD1 interactome was therefore conducted at 3 Zeitgeber times (ZT0, 9, and 16) under the long-day (16-h L/8-h D) condition (Fig. 2*A*, details in *Materials and Methods*). Label-free quantification (LFQ) approach with Cycloess normalization was used to improve the quantification of biotinylated peptides by minimizing batch variations and maximizing data correlations (Dataset S1 and *SI Appendix*, Fig. S2).

A total of 219 candidate LWD1-interacting proteins were enriched in LWD1-TbID, including 190, 104, and 84 proteins identified at ZT0, 9, and 16, respectively (Fig. 2 *B*–*D* and Dataset S2; selection flowchart see *SI Appendix*, Fig. S3). Among them, TCP22 was highly enriched at all ZTs examined (Fig. 2 *B*–*D* and Dataset S2), consistent with the LWD1–TCP22 complex formation at multiple time points during the day (9, 17). In addition to TCP22, approximately 20% of the LWD1 interactome were identified at all 3 ZTs (*SI Appendix*, Fig. S4 *A* and *B* and Dataset S2), including known clock proteins TIME FOR COFFEE (TIC), EARLY FLOWERING 3 (ELF3), PRR7, and SNW/SKI-INTERACTING PROTEIN (SKIP) (Fig. 2 *B*–*D* and Dataset S2). Among them, the clock protein ELF3 was previously shown to interact with LWD1 (18).

An AI-based structure prediction program AlphaFold-Multimer (19) was applied to evaluate the robustness of our TurboID approach by calculating the interaction probability between LWD1 and the 219 candidate proteins. By ranking the interface predicted template modeling (ipTM) scores, more than 65% of these candidates were predicted to have positive interactions with LWD1 when applying a criterion of ipTM score ≥0.5 (20) (*SI Appendix*, Fig. S5*A* and Dataset S3). Those with higher ipTM scores also tend to have significantly higher enrichment of biotinylation levels in LC–MS/MS results (*SI Appendix*, Fig. S5*B*).

Interestingly, some of the 219 candidate LWD1-interacting proteins belong to paralogous groups with members previously identified as clock proteins (21–24). These include transcriptional coregulators, SIN3-like proteins (SNL3 and SNL4), and TOPLESS-related proteins (TPL and TPR2). In addition, LWD1 also interacts with transcriptional factors such as homeobox proteins (HB23, HB30, and HB34) and PHD-finger proteins (TTA1 and TTA2) (Dataset S2). None of the homeobox and PHD-finger proteins have previously been reported as clock components. By bimolecular fluorescence complementation (BiFC) assays, we confirmed the protein–protein interaction between LWD1 and HB23/TTA2 (*SI Appendix*, Fig. S6), two proteins that highly enriched in LWD1-TbID at multiple time points (Dataset S2).

Gene ontology (GO) analyses of the LWD1 interactome revealed overrepresentation in functions related to circadian rhythm, chromosome organization, and RNA processing (Fig. 2*E* and *SI Appendix*, Fig. S4*C* and Dataset S4), consistent with the transcriptional and cotranscriptional regulations imposed by circadian clock (25, 26). The functional diversities of the identified LWD1-interacting proteins also suggest that, in addition to being a transcriptional coactivator (9), LWD1 may also contribute to epigenetic and posttranscriptional regulations of clock gene expression.

### LWD1-Interacting Epigenetic and Transcriptional Regulators Are New Clock Proteins.

**Fig. 3.**
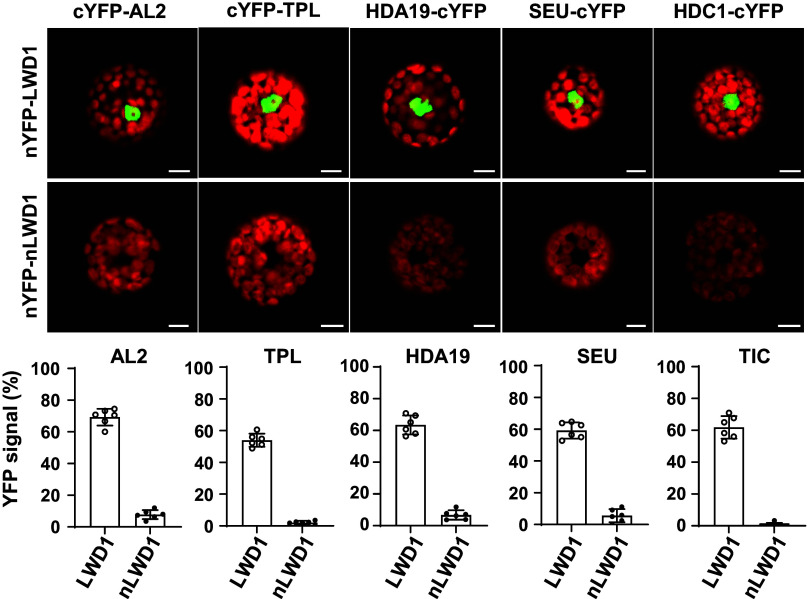
Interactions between LWD1 and epigenetic regulators validated by BiFC assay. cYFP-fused AL2, TPL, HDA19, SEU, or HDC1 were coexpressed with nYFP-fused LWD1 or nLWD1 (negative control) in *Arabidopsis* protoplasts. Yellow fluorescent protein (YFP) signals (in green) indicated interactions between LWD1 and its candidate partners. Representative images are shown (*Top* panel). The percentage of protoplasts displaying nuclear YFP signals in experimental (LWD1) and control (nLWD1) was calculated (n ≥ 200 protoplasts in each of the 3 biological replicates). Data are presented as means with SD. (Scale bar, 10 μm.)

Several prior studies reported pivotal roles of epigenetic regulators in modulating the expression of core clock genes (25, 27, 28). We first used BiFC assays to confirm that LWD1 interact with several epigenetic regulators, including ALFIN-LIKE 2 (AL2), TPL, HISTONE DEACETYLASE 19 (HDA19), SEUSS (SEU), and HISTONE DEACETYLATION COMPLEX 1 (HDC1) (Figs. 2*E* and 3). We further examined the protein–protein interaction between LWD1 and the epigenetic regulator TPL and SEU by using yeast two-hybrid (Y2H) assays (*SI Appendix*, Fig. S7). Those not validated by Y2H likely involve transient or weak interactions, require posttranslational modifications, or additional partner proteins that are absent in yeast (29, 30). Also, the bona fide interactor TPL was assigned a low ipTM score by AlphaFold-Multimer (19) (*SI Appendix*, Fig. S5*A*), highlighting that our TurboID approach offers a more robust and sensitive identification of LWD1-associated proteins than current computational predictions alone.

To assess the roles of these LWD1-interacting epigenetic regulators and the transcriptional factors HB23 and TTA2 (*SI Appendix*, Fig. S6) in the *Arabidopsis* circadian clock, the reporter gene *pCCA1:LUC2* (12) was introduced via genetic crossing into *Arabidopsis* mutants defective in the epigenetic regulators, including *al2*, *tpl*, *tpl/tpr1/tpr4* triple mutant (*tplT*), *hda19*, *seu,* and *hdc1*, and transcription factors, *hb23* and *tta2*. The *pCCA1:LUC2* gene allowed us to monitor how the mutations of these genes impact the circadian period length. Results showed that two independent *seu* mutants (*seu-1* and *seu-2*) displayed significantly shorter period lengths and this defect can be restored in the *seu-1 SEU-FLAG* complementation lines (*SI Appendix*, Fig. S9). These findings support the role of SEU, a LWD1-interacting epigenetic regulator, as a previously undescribed regulator of circadian clock in *Arabidopsis*.

**Fig. 4.**
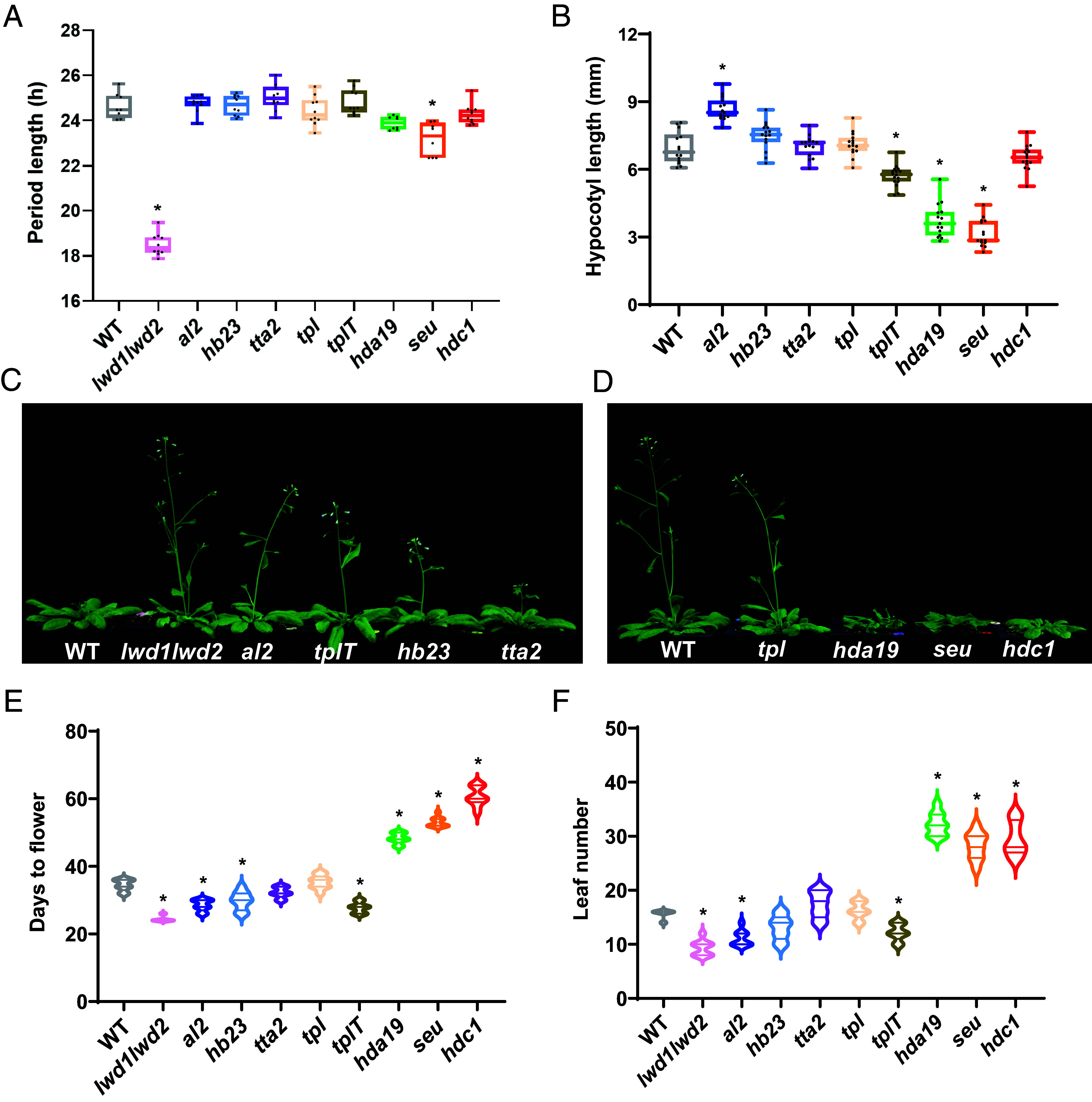
Phenotypic characterizations of the LWD1-interacting epigenetic regulator mutants. (*A*) Circadian periods of WT, *lwd1lwd2*, *seu*, *hda19*, *tpl*, *tplT*, *al2*, *hb23*, *hdc1,* and *tta2* mutants carrying *pCCA1:LUC2* reporter gene. Seedlings (n ≥ 8) were entrained under a 16-h light and 8-h dark cycle for 7 to 9 d and transferred to continuous white light (LL, 36 μmol m^−2^s^−1^) to assess circadian period with 95% confidence according to LL48 to LL120 using FFT-NLLS analysis. Boxes show medium values and 25 to 75 percentile range and whiskers indicating the minimum to maximum range. (*B*) Bar graph showing hypocotyl lengths of 4-d-old light-grown (10 μmol m^−2^s^−1^) WT and mutant seedlings (n ≥ 10). (*C*) Image showing flowering phenotype of WT, *lwd1lwd2*, *al2*, *tplT*, *hb23*, and *tta2* plants grown in long day (16-h light and 8-h dark) condition for 24 d. (*D*) Image showing flowering phenotype of WT, *tpl*, *had19*, *seu*, and *hdc1* plants grown in long day condition for 36 d. (*E* and *F*) Flowering time of WT, *lwd1lwd2,* and epigenetic regulator mutants was represented by days to flower (*E*) or leaf number (*F*). At least 9 plants for each genotype were used in each individual experiment. Violin plots showed the minimum to maximum range and bars indicating mean, 25, and 75 percentile range. Significance difference relative to WT was assessed using one-way ANOVA with Dunnett’s multiple comparison test in (*A*, *B*, *E*, and *F*). Three independent biological experiments were performed with similar results. *P-value ≤ 0.01.

In contrast, mutations in other LWD1-interacting candidates, despite their interactions with LWD1 via BiFC or Y2H assays (Fig. 3 and *SI Appendix*, Figs. S6 and S7), did not exhibit notable changes in circadian period (Fig. 4*A* and *SI Appendix*, Fig. S8). This lack of phenotype may be attributed to functional redundancy among gene family members, particularly for AL2, HB23, TTA2, and TPL, as listed in Dataset S2. To circumvent this, we generated transgenic lines overexpressing HB23 (*HB23-3HA*), a repressor fusion of HB23 (*HB23-SRDX*), and an activator fusion (*HB23-VP16*) in *Arabidopsis* carrying the reporter gene *pCCA1:LUC2*. Results showed that both *HB23-3HA* and *HB23-SRDX* lines exhibited shorter circadian period lengths, whereas *HB23-VP16* lines showed longer period lengths (*SI Appendix*, Fig. S10). These results indicated that HB23 regulates the *Arabidopsis* circadian clock, likely by acting as a transcriptional repressor.

*Arabidopsis* circadian clock is known to regulate numerous developmental processes, including the light-inhibited hypocotyl elongation and the developmental transition from vegetative to reproductive development (flowering) (5, 13, 31). We compared both the hypocotyl elongation and the flowering time phenotypes of these epigenetic and transcription factor mutants with WT plants. When young seedlings were grown in the dark for 4 d (skotomorphogenic development), all but *hdc1* epigenetic mutants exhibited comparable hypocotyl length with that of WT (*SI Appendix*, Fig. S11), suggesting hypocotyl cells in these mutants (except for *hdc1*) have normal elongation rates. However, when grown in the light for photomorphogenic development, *al2* mutant seedlings exhibited light-hyposensitive phenotype with longer hypocotyls, while the *tplT, hda19*, and *seu* mutant seedlings are light hypersensitive with shorter hypocotyls (Fig. 4*B*). We further investigate whether these LWD1-interacting proteins regulate hypocotyl growth via the clock output pathway involving PIF4 and PIF5 (32–34). Results indicated the expression of *PIF4* and *PIF5* at ZT3-6 was not significantly correlated with the hypocotyl phenotypes observed (*SI Appendix*, Fig. S11), indicating that these LWD1-interacting proteins regulate hypocotyl elongation via a mechanism distinct from the clock-PIF module.

We also recorded the flowering time of these mutants by both the time and the leaf number at bolting. Results showed that *al2, hb23,* and *tplT* displayed early-flowering phenotype compared to WT, whereas *hda19*, *seu,* and *hdc1* mutants were late flowering (Fig. 4 *C*–*F*). To elucidate which pathway is responsible for the flowering phenotype in the mutants, the expression levels of flowering regulators, including CO/FT in the photoperiodic pathway, FLC in the vernalization and autonomous pathways, and LFY in the gibberellin pathway (35), were measured at ZT15-16. The results show that flowering time in these mutants is negatively correlated with *CO* and *FT* expression levels (*SI Appendix*, Fig. S12 *A* and *B*), whereas there is no correlation with *FLC* or *LFY* (*SI Appendix*, Fig. S12 *C* and *D*). These results imply that these LWD1-interactor mutants likely have clock defects that lead to aberrant photoperiodic outputs and changes in flowering time.

Taken together, the above data indicated these epigenetic and transcriptional regulators contribute to at least one of the developmental processes controlled by the circadian clock in *Arabidopsis*. The results also corroborated the throughput and robustness of the TurboID-based profiling of LWD1-interactome in revealing new players in the *Arabidopsis* circadian clock and circadian regulated downstream developmental processes.

### LWD1 Is a Corepressor Working With PRR5 and TOC1 to Repress *CCA1* Expression.

**Fig. 5.**
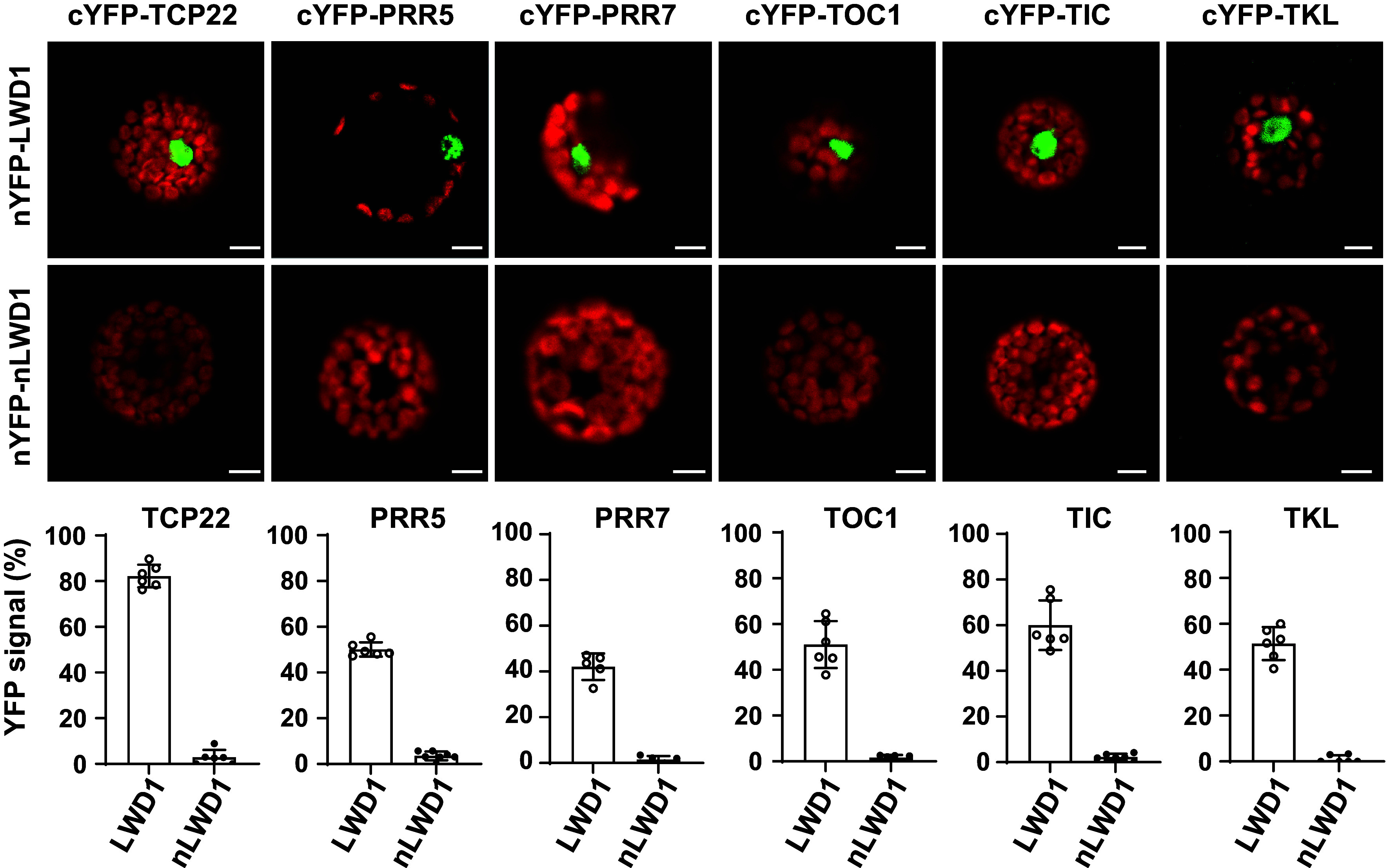
Interactions between LWD1 and key clock components validated by BiFC. cYFP-fused TCP22, PRR5, PRR7, TOC1, TIC, and TKL were coexpressed with nYFP-fused LWD1 or nLWD1 in *Arabidopsis* protoplasts. Yellow fluorescent protein (YFP) signals (in green) indicated interactions between LWD1 and its candidate partners. Representative images are shown (*Top* panel). The percentage of protoplasts displaying nuclear YFP signals in experimental (LWD1) and control (nLWD1) was calculated (n ≥ 200 protoplasts in each of the 3 biological replicates). Data are presented as means with SD. (Scale bar, 10 μm.)

Key circadian clock components, such as ELF3, PRR5, PRR7, TOC1, TIC, and TKL, were identified as LWD1-interacting proteins and marked with high probabilities of forming complexes with LWD1 (ipTM score greater than 0.8; *SI Appendix*, Fig. S5*A*). We experimentally confirmed interactions between LWD1 and PRR5, PRR7, TOC1, TIC, and TKL with TCP22 included as a positive control (Fig. 5). The interactions of LWD1 with the PRR7, PRR5, and TOC1 were also confirmed by Y2H and in vitro pull-down assays (*SI Appendix*, Figs. S7 and S13). Our data support that LWD1 acts as a hub for recruiting multiple clock proteins at different times throughout the day.

**Fig. 6.**
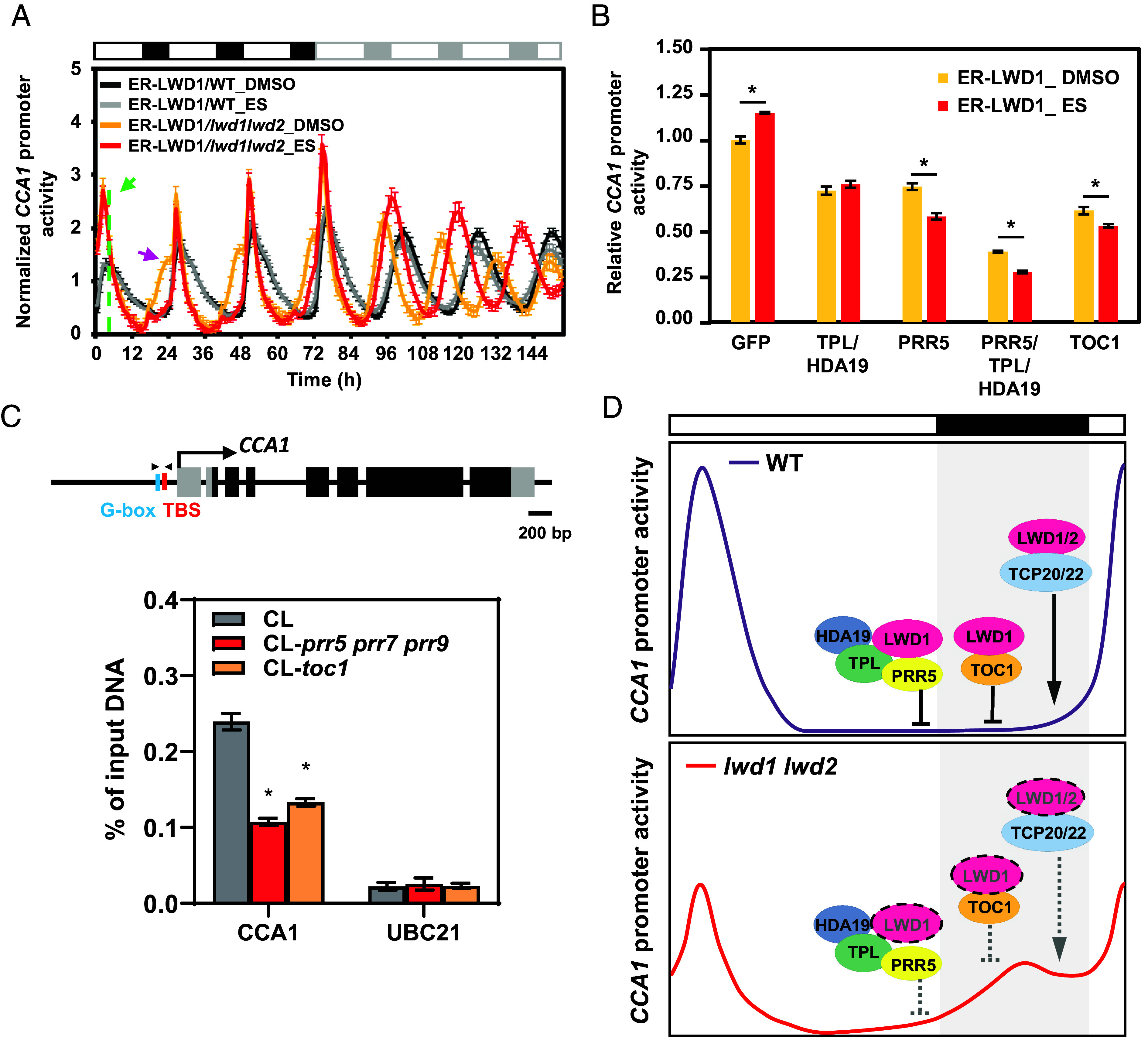
LWD1 forms a complex with PRRs, TPL, and HDA19 to sustain the trough expression of *CCA1* in the late afternoon and early evening. (*A*) The normalized *pCCA1:LUC2* activities in WT or *lwd1lwd2* carrying *pER:LWD1-EYFP-HA* were monitored under long-day conditions (16-h light/ 8-h dark) for 3 d and followed by continuous light treatment for 3 d. LWD1 expression was induced by 20 μM β-estradiol (ES) at ZT4 of day one (green arrowhead; ES), with DMSO treatment as the control (DMSO). The purple arrow marks the advanced *CCA1* expression peak in DMSO-treated *lwd1lwd2* mutant. White, black, and gray bars denote the light, dark, and subjective darkness, respectively. Similar results were observed in three independent experiments. Data are means with SD (n ≥ 10). (*B*) *Arabidopsis* protoplasts isolated from *pER:LWD1-YFP lwd1lwd2* plants carrying *pCCA1:LUC2* were transfected with combinatorial plasmids marked at ZT11, treated with 10 μM β-estradiol (ES) or DMSO at ZT12 and samples collected at ZT18 for bioluminescence assays. Values shown were normalized to *35S:GFP* control transfection treated with DMSO. Data (n = 4) are mean ± s.d. Three biological replicates had similar results. * indicate Student’s *t* test *P* value < 0.001. (*C*) The binding of LWD1 to *CCA1* promoter in the early evening is reduced in *prr5 prr7 prr9* and *toc1* mutants. The diagram shows the gene structure of *CCA1*. The transcriptional start site and exons are marked with an arrow and boxes. The solid black boxes and gray boxes represent the coding region and untranslated regions, respectively. Two arrowheads showed the forward and reverse primers for amplifying fragments containing the TOC1/PRR binding sites harboring G-box and TCP-binding site (TBS) (40, 41). *UBC21* was utilized as a negative control locus. Data are presented as mean ± SD (n =4 technical replicates). * indicates Student’s *t* test *P* value < 0.01. Results are representative of four independent biological experiments. (*D*) A diagram illustrates dual functions of LWD1 by interacting with TCP20/TCP22 to activate *CCA1* expression at dawn, and interacting with PRR proteins to repress *CCA1* expression at late afternoon and early evening.

These results prompted us to assess the biological functions of LWD1 in the afternoon and early evening when PRR7, PRR5, and TOC1 proteins primarily accumulated (36–38) and were demonstrated to repress the expression of *CCA1* (39, 40). By measuring the promoter activities of *CCA1* under diel entrainment and constant light conditions, an expression peak of *CCA1* at dawn (and subjective dawn) was observed in WT seedlings (Fig. 6*A*). Under diel entrainment conditions, two *CCA1* expression peaks were observed in *lwd1lwd2* seedlings: a light-induced peak at dawn which is consistent with that in WT seedlings and the derepression of *CCA1* starting from late afternoon and reaching the second expression peak in the early night (Fig. 6*A* and *SI Appendix*, Fig. S14 *A* and *B*).

Whether the afternoon/evening *CCA1* expression peaks under diel entrainment condition was a result of *CCA1* derepression in the absence of LWD1/LWD2 in *lwd1lwd2* mutant was next investigated. We generated transgenic lines expressing *pER*:*LWD1-EYFP-HA* in both WT and *lwd1lwd2* mutant carrying *pCCA1:LUC2* reporter gene, allowing for testing the impact of controlled induction of LWD1 by estradiol (ER in Fig. 6) on the expression behavior of *CCA1*. Results showed that, when *LWD1-EYFP* was induced at ZT4, the afternoon/evening *CCA1* expression peak was reduced subsequently (Fig. 6*A* and *SI Appendix*, Fig. S14*B*) and the induced expression of *LWD1-HA-EYFP* can rescue the short-period phenotype in *lwd1lwd2* (*SI Appendix*, Fig. S15). In contrast, there was no noticeable effect in the DMSO-treated *lwd1lwd2* seedlings (Fig. 6*A*), or in estradiol- and DMSO-treated WT seedlings (Fig. 6*A*). These results suggested that LWD1 contributes to the *CCA1* repression in the late afternoon/early evening.

We hypothesize that LWD1 represses the *CCA1* expression in the late afternoon/early evening by interacting with established repressors, including PRR5 and TOC1, and the TPL–HDA19 trimeric complex identified in this study (Figs. 3 and 5 and Dataset S2) (23, 39, 40). To test this, we examined the impact of transient LWD1 induction on *CCA1* promoter activity in *lwd1lwd2* mutant in the combinatorial presence of PRR5, TOC1, and the TPL–HDA19 complex. The result demonstrated that the induction of *LWD1* expression effectively facilitates the repressor functions of PRR5, PRR5–TPL–HDA19, and TOC1 on *CCA1* expression (Fig. 6*B*). Furthermore, we performed ChIP-qPCR to determine if PRRs and TOC1 are required to recruit LWD1 to the *CCA1* promoter. Results showed that the LWD1 occupancy at the *CCA1* promoter was significantly attenuated in *prr5prr7prr9* and *toc1* mutants, compared to that in the *LWD1-FLAG* control line at ZT16 (Fig. 6*C*). These results imply that the PRR/TOC1 repressor proteins interact with LWD1, which lacks DNA-binding domains (13), to facilitate its association with *CCA1* promoter.

Taken together, our data supported that in addition to the coactivator role of LWD1 for boosting the *CCA1* expression at dawn (9), LWD1 also interacts with the PPR-TPL-HDA protein complexes and TOC1 to suppress the expression of *CCA1* from late afternoon to early evening in WT *Arabidopsis* under the diel environment (Fig. 6 *D*, *Upper* panel). This is consistent with the association of LWD1 with the *CCA1* promoter at night (9). The consecutive functions of LWD proteins first as a coactivator and then a corepressor through working with different protein partners together shape the expression pattern of *CCA1* during a 24-h day. In *lwd1lwd2* mutants, the lack of LWD proteins leads to early derepression of *CCA1* in the afternoon/evening and compromised activation of *CCA1* at dawn, resulting in advanced expression of *CCA1* with reduced oscillating amplitude (Fig. 6 *D*, *Lower* panel).

## Discussion

The employment of the TurboID-based proximity labeling in this study successfully identified known and new LWD1-interacting protein partners of functional diversities. This approach is highly complementary to the traditional forward genetic screening, particularly for identifying functionally redundant and/or paralogous proteins (Dataset S2 and *SI Appendix*, Fig. S10). Nevertheless, achieving a truly comprehensive LWD1-interactome profile can be benefited by a broader temporal sampling across more ZTs and overcoming the current technical constraints of proximity labeling (42) and mass spectrometry.

The formation of diverse protein complexes involving LWD1 is crucial to sustaining a precise and fully functional circadian clock in *Arabidopsis*, explaining the dramatic reductions in the clock period length and amplitude in *lwd1lwd2* mutant (12, 13). By forming protein complexes with transcription factors, epigenetic remodelers, and splicing factors, LWD1 likely has a broader role at the transcriptional and posttranscriptional levels of gene expression regulation (Fig. 2*E* and *SI Appendix*, Fig. S4*C* and Datasets S2 and S4).

We identified a LWD1-interacting protein as new clock proteins for maintaining the period length of the *Arabidopsis* clock. Notably, mutant defective in *SEU* has a short period length (Fig. 4*A* and *SI Appendix*, Fig. S9). Given the functional redundancy between SEU and its paralogs, SEUSS-LIKE proteins (SLKs), in regulating plant development (43), a higher-order *seu slk* mutant may exhibit enhanced clock defects. Additionally, SEU was previously shown to form corepressor complexes with LEUNIG (LUG) and LEUNIG _ HOMOLOG (LUH) for the regulation of embryonic and floral development (44). The LWD1 interactome includes SEU, LUG, LUH, and LUG interacting HDA19 (45) (Fig. 2*E* and Dataset S2), implying a corepressor complex composed of LWD1, SEU, LUG/LUH, and HDA19 in regulating clock gene expression via histone modification and chromatin remodeling.

Previous studies have shown that *CCA1* expression aligns with daily changes in histone H3 acetylation in the chromatin region (9, 39, 40, 46–48). By recruiting epigenetic modifiers such as histone deacetylases (HDAs) or histone acetyltransferases (HACs) (Dataset S2), LWD1 could conceivably function as a coactivator (9) or corepressor (Fig. 6) to dynamically modulate the chromatin status of *CCA1*, ensuring that gene expression is timely and precisely regulated during the 24-h diel cycle. Indeed, our findings revealed that LWD1 forms repressive complexes with PRR-TPL and TOC1 to suppress *CCA1* in the late afternoon and early evening (Figs. 3, 5, and 6). In addition to regulating *CCA1* expression, PRR5 and TOC1 also play broader roles in regulating additional clock genes, such as *LATE ELONGATED HYPOCOTYL, REVEILLEs, NIGHT LIGHT–INDUCIBLE AND CLOCK-REGULATEDs*, and growth-related genes (40, 49, 50). How LWD1 contributes to the full functions of PRR5 and TOC1 in the broad gene expression regulation warrants further investigations.

## Materials and Methods

Detailed information on plant material and growth conditions, generation of constructs and transgenic lines, protein analyses (isolation and LC–MS/MS analyses of biotinylated peptides, immunoblot, Y2H and in vi*tro* pull-down assays), cell biology experiments (subcellular localization and BiFC assays), phenotypic assessments (flowering time and hypocotyl length measurement), data processing and bioinformatic analyses, nucleic acid analyses (ChIP-qPCR, RNA isolation, and qRT-qPCR assays), bioluminescence measurement and dual luciferase assays are provided in *SI Appendix*, *Materials and Methods*. The primers used in this study are listed in Dataset S5.

## Supplementary Material

Appendix 01 (PDF)

Dataset S01 (XLSX)

Dataset S02 (XLSX)

Dataset S03 (XLSX)

Dataset S04 (XLSX)

Dataset S05 (XLSX)

## Data Availability

The main figures and the Extended Data included data needed to evaluate the conclusions drawn. Source data are provided with this paper. Plant and plasmid materials are available upon request. The mass spectrometry proteomics data have been deposited to the ProteomeXchange Consortium via the PRIDE partner repository with the project accession: PXD062258 (51).
