# Supplementary material for: Unveiling previously undescribed circadian clock regulators and action mechanisms via TurboID-based profiling of the LWD1 interactome in Arabidopsis thaliana
Source: Proc Natl Acad Sci U S A. 2026 Aug 5;123(32):e2618203123. doi: 10.1073/pnas.2618203123 (PMC13461971; doi:10.1073/pnas.2618203123)
Supplement: Supplementary file 1 [file pnas.2618203123.sapp.pdf]

Supporting information for

Unveiling previously undescribed circadian clock regulators and action mechanisms via TurboID-based profiling of the LWD1 interactome in *Arabidopsis thaliana*

Authors:

Chun-Kai Huang[1], Chuan-Chih Hsu[1], Huang-Lung Tsai[2], Wen-Dar Lin[1], Jing-Fen Wu[1] and Shu-Hsing Wu[1*]

Affiliations:

[1]Institute of Plant and Microbial Biology, Academia Sinica, Taipei 115201, Taiwan
[2]Institute of Molecular and Cellular Biology, National Taiwan University, Taipei 10617, Taiwan

Correspondence to: Shu-Hsing Wu
Email: shuwu@as.edu.tw

This PDF file includes:
Supporting Materials and Methods
Fig. S1 to S15
SI References

Other supporting materials for this manuscript include the following:
Dataset S1-S5

SI Appendix, Materials and Methods.

Plant material and growth conditions

This study used the wild-type *Arabidopsis thaliana* ecotype Columbia-0 (Col-0; WT), *lwd1lwd2* (1), *pCCA1:LUC2* (2), and *lwd1lwd2 pCCA1:LUC2* reporter lines (2). The epigenetic mutants, *seu-1* (Salk_069303), *seu-2* (Salk_120524), *tpl* (Salk_097230), *tpl/tpr1/tpr4* triple mutant (*tplT*) (CS72353), *hb23* (Salk_059288), *al2* (Sail_663_E04), *hdc1* (GABI_054G03), and *tta2* (Sail_323_D07) were obtained from the Arabidopsis Biological Resource Center. The clock mutants used were *toc1-101* (*toc1*)(3), *prr5-11* (*prr5*), *prr7-11* (*prr7*)(4), and *prr9-10* (*prr9*)(5). Seeds were surface-sterilized and evenly distributed on half-strength Murashige and Skoog (MS) (Duchefa) medium plates containing 0.8% phyto agar (Duchefa), pH 5.7. Following a four-day stratification in the dark at 4°C, the seeds were transferred to growth chambers for growth at 22°C and illuminated with 80-100 µmole $m^{-2}s^{-1}$ of white light.

Generation of *LWD1-TurboID* and *nLWD1-GFP-TurboID* transgenic plants

To generate *pLWD1:LWD1-GS-HA-TurboID* (*LWD1-TbID)* and *pLWD1:nLWD1-GFP-GS-HA-TurboID* (*nLWD1-TbID)* plasmids, *pLWD1:LWD1* (-1068 to +1038, relative to translation start site) and *GS-HA-TurboID* fragments were amplified by polymerase chain reaction (PCR) using Arabidopsis genomic DNA and plasmid *HA-TurboID-NLS_pCDNA3* (6) (Addgene, #107171) as templates with specific primers (listed in **Dataset S5**), respectively. The three partially overlapping fragments, *pLWD1:LWD1, GS-HA-TurboID* and *Pst*I-*Sac*I digested *pCAMBIA1390*, were fused via homologous recombination with UltraFast recombinase mix (Tools) following the manufacturer's protocol to obtain *pLWD1:LWD1-GS-HA-TurboID* plasmid. For *pLWD1:nLWD1-GFP-GS-HA-TurboID* plasmid, *pLWD1:nLWD1* (-1068 to +189) and *enhanced green fluorescent protein (EGFP)* fragments were amplified by PCR from Arabidopsis genomic DNA and *p35S:EGFP*, respectively. The four fragments, *pLWD1:nLWD1* (-1068 to +189), *eGFP*, *GS-HA-TurboID* and *Pst*I-*Sac*I digested *pCAMBIA1390* with overlapping sequences were fused by homologous recombination as stated above to obtain *pLWD1:nLWD1-GFP-GS-HA-TurboID* plasmid. *pLWD1:LWD1-GS-HA-TurboID* and *pLWD1:nLWD1-GFP-GS-HA-TurboID* plasmids were transformed separately into *lwd1lwd2* double mutant to

generate stable transgenic *pLWD1:LWD1-GS-HA-TurboID/lwd1lwd2* and *pLWD1:nLWD1-GFP-GS-HA-TurboID/lwd1lwd2* lines using *Agrobacterium*-mediated floral dip method (7) with *Agrobacterium tumefaciens* strain GV3101.

Immunoblot analyses

Total proteins from transgenic lines or protoplasts transiently expressing heterologous proteins were extracted by SDS sample buffer and subjected to immunoblot analyses (8). LWD1-specific antiserum (9), anti-HA antiserum (Sigma, H3663) and anti-FLAG (Sigma, F1804) were used as primary antisera. Horseradish peroxidase (HRP)-conjugated streptavidin (Jackson ImmunoResearch, 016-030-084) were used for the detection of biotinylated TCP22-FLAG.

Subcellular localization of nLWD1-eGFP

The *pLWD1:LWD1-GFP* (2) or *pLWD1:nLWD1-GFP-GS-HA-TurboID* were transfected into Arabidopsis protoplasts as previously described (10) by adopting a sandwich method (11). GFP signals were observed using confocal microscopy (LEICA STELLARIS 8).

Flowering time determination

The flowering time of Arabidopsis mutants and transgenic lines indicated by both the days to flower and rosette leaf numbers were recorded when the primary inflorescence reached ~3 cm long. Each measurement was repeated at least three times under long-day conditions (16 h light/ 8 h dark) illuminated with 80-100 µmole $m^{-2}s^{-1}$ of white light.

Isolation of biotinylated peptide

Approximately 1 g rosette leaves of 14-d-old Arabidopsis seedlings from Zeitgeber time (ZT)0, ZT9, and ZT16 grown on 1/2 MS solid medium under long-day conditions were collected. The collected samples were immediately submerged in 1/2 MS medium with

50 µM biotin and subjected to a vacuum of -200 mmHg for 5 min and returned to the long-day growth chamber to undergo biotinylation reactions for 1 h.

Excess media was removed from the plant tissues with paper towel before they were ground into powders in liquid nitrogen. The ground powder samples were suspended in cold nuclei isolation buffer (NIB) [40 mM MES-KOH pH 5.7, 0.8 M sucrose, 10 mM EDTA, 50 µM MG132, and 50x diluted (v/v) protease inhibitor cocktail (Roche)]. The crude extracts were sequentially filtered through 100 µm and 40 µm nylon meshes, after which the filtered homogenates were centrifuged at 1,500 x g at 4 °C for 10 min to pellet the nuclei. These crude nuclei samples were then resuspended in NIB solution containing 0.3% Triton X-100 for nuclei preparation as described in the plant nuclei extraction kit (Sigma). The nuclear proteins were extracted from nuclei pellets and resuspended in 300 µL of nuclear lysis buffer [50 mM Tris-HCl (pH 7.5), 750 mM NaCl, 1 mM EDTA, 5 mM DTT, 1% Triton X-100, 0.5% SDS, 10 µM MG132, and 50x diluted (v/v) protease inhibitor cocktail (Roche)] by sonication in an ice bath with 10 cycles (30 sec ON/1 min OFF) on high-strength conditions using a Bioruptor UCD-200 (Diagenode). The nuclear proteins in the supernatants were collected by centrifugation at 12,000 × g for 10 min.

Extracted nuclear proteins were deoxidated and alkylated using 10 mM tris-(2-carboxyethyl)-phosphine, hydrochloride (TCEP) and 40 mM chloroacetaldehyde (CAA) at 45°C for 15 min. A final concentration of 5.5% (v/v) phosphoric acid followed by 6-fold volume of binding buffer (90%, v/v, MeOH in 100 mM triethylammonium bicarbonate buffer (TEAB), pH 8.5) were added sequentially to the above protein solution. After gentle vertexing, the solution was cleaned up by using a suspension trapping (S-trap) micro-column (Protifi) as described (12) and digested into peptides by adding a mixture of trypsin (Promega) and Lys-c endopeptidase (Wako) into the S-Trap micro column with enzyme-to-total protein ratios of 1 µg:50 µg and 1 µg:200 µg, respectively. The digestion was conducted at 37°C for 2 h with agitation at 750 rpm. Following digestion, peptide samples were further purified via an Oasis column, following standard procedures (Waters), to remove proteases and the peptides were eluted using 500 µL of 1x Phosphate-buffered saline (PBS). The purified peptides were then incubated with 100 µL of Dynabead M-280 Streptavidin beads (Invitrogen, 11205D), which had been pre-washed with 1x PBS, for 4 h at 4°C. After incubation, the beads were sequentially washed with 1 mL of 1x PBS containing 5% acetonitrile (ACN)

and ddH2O with 5% ACN, each for 5 min with shaking at 800 rpm at room temperature. The biotinylated peptides were then eluted using 200 µL of preheated (65°C) elution buffer, comprising 0.2% trifluoroacetic acid (TFA), 0.1% formic acid (FA), and 80% ACN, at 65°C for 5 minutes, repeated three times. The eluted biotinylated peptide samples were collected and dried using a vacuum concentrator and dissolved in 200 µL 5% ACN and 0.1% TFA solution and desalted with two layers of SDB-XC StageTips (3M). Lastly, the enriched biotinylated peptides were eluted with 20 µL of 5% ACN and 0.1% TFA solution and dried using a vacuum concentrator.

LC-MS/MS analysis and biotinylated peptide identification

Dried biotinylated peptide samples were dissolved in 10 µL of 0.1% FA with 3% ACN, and 4 µL of the sample was injected into an Easy-nLC 1200 system coupled with an Orbitrap Fusion Lumos Tribrid mass spectrometer (Thermo Fisher Scientific). Buffer A was 0.1% FA, and buffer B was 0.1% FA in 80% ACN. The LC gradient over 120 min was established from 5% to 45% buffer B at a flow rate of 300 nl/min. Peptides were separated on a 25 cm PepMap C18 column (Thermo Fisher Scientific) with a column heater set at 45°C. The mass spectrometer was operated in the data-dependent acquisition mode, in which a full MS scan (m/z 350–1,600; resolution: 240,000) was followed by the most intense ions being subjected to higher-energy collision-induced dissociation (HCD) fragmentation within 3 sec cycles. Data were acquired in the ion trap [normalized collision energy (NCE): 30; max injection time: 100 ms; isolation window: 1.6 m/z; dynamic exclusion: 15 s].

All mass spectrometry files were analyzed using Proteome Discoverer (version 2.5.0.400) with SequestHT and Mascot search engines against the TAIR10 database (35,386 entries or 70,774 entries with 50% decoys; version: 2019-07-11). Peptide spectrum identification was conducted with the precursor mass tolerance set to ±10 ppm, and fragment mass tolerance set to 20 ppm. Protein digestion was set to trypsin (after KR/-) and allowed two missed cleavages. The fixed modification was set as carbamidomethyl, and variable modifications were set as oxidation at methionine, acetylation in protein N-terminal, and biotinylation at lysine for peptide recognition. The cutoff of peptide spectrum match (PSM), peptide, and protein level was set with a false discovery rate of 1%. Label-free quantification was performed using the nodes

of Minora feature detector and precursor ion quantifier embedded in Proteome Discoverer.

Data processing and bioinformatic analyses

Quantifiable intensity values for LFQ were processed using NormalyzerDE (13) (http://quantitativeproteomics.org/normalyzer) and the CycLoess method that has the lowest pooled coefficient of variation and the highest Spearman correlation across the three biological replicates at each ZT point was used for LFQ intensities normalization from triplicate replicates. To calculate the normalized intensities in $\log_2$ scale, the imputation was done by replacing values missing in all three replicates with the average of the lowest 5% normalized intensities minus 1 in the corresponding control datasets. The adjusted normalized intensity values were processed using NormalyzerDE for differential expression analyses using the empirical Bayes Limma approach (14). Significantly enriched biotinylated peptides were identified if -log *p*-values ≥ 1.301 and the fold change $\log_2$(LWD1-TbID/nLWD1-TbID) ≥ 2. The -log *p*-values and $\log_2$ fold change were used for generating scatter plots shown in **Fig. 2**.

Gene Ontology (GO) enrichment analysis was performed using Gene Ontology Browsing Utility (15) with Fisher's exact test ≤ 1.0×10^{-3} as the cutoff, with annotation from the TAIR10 database. The protein interaction network analysis of the biotinylated proteins identified was performed by using the String database (16) (https://string-db.org/network/ 9606.ENSP00000421736) (version 11.5) with a high confident interaction score set at ≥ 0.7 as the cutoff. Protein–protein interaction networks were visualized using Cytoscape (17) (version 2.7.2).

Bimolecular fluorescence complementation (BiFC) assay

For BiFC assay, *pSAT4-nEYFP*-N1 (18) was modified to generate *pNOS promoter* (*pNOS*):*nEYFP-LWD1-35S* terminator (*35St*), and *pSAT4-cEYFP-N1* (18) to *pNOS*:*cEYFP-MCS* (*Apa*I-*Pac*I)-*35St* or *pNOS*:*MCS* (*Asc*I-*Avr*II-*Cla*I)-*cEYFP-35St*. PCR was used to amplify desirable DNA fragments with specific primer pairs shown in **Dataset S5** and the fragments were assembled by UltraFast recombinase mix (Tools). The full-length coding sequences of LWD1 interacting targets, such as *TCP22*, *PRR5*, *PRR7*, *TOC1*, *TIC*,

TKL, *AL2*, *HB23*, *TPL*, *HDC1*, *SEU*, *HDA19* and *TTA2,* were cloned into *pNOSp*:*cEYFP-MCS* (*Apa*I-*Pac*I)-*35St* or *pNOSp*:*MCS* (*Asc*I-*Avr*II-*Cla*I)-*cEYFP-35St* and in-frame with *cEYFP*. *Not*I-digested fragments of above clones were subcloned into *Not*I-digested *pNOS*:*nEYFP-LWD1-35St*. Finally, plasmids expressing the BiFC interacting pairs were used for transient assays in Arabidopsis protoplasts with *pNOS-nEYFP-nLWD1* as the negative control. Briefly, 10 µg of plasmids were transformed into fresh prepared protoplasts and the yellow fluorescence signal (YFP) was observed under by LEICA STELLARIS 8 confocal microscopy using excitation 514 nm and emission at 535-560 nm with TauGating from 1 ns to 10 ns. The ratio of protoplasts (n ≥ 200) with or without complementary YFP fluorescence signal were calculated as shown in **Fig. 3 and 5**.

In silico **protein-protein interactions prediction**

AlphaFold-Multimer was used to assess the probabilities of protein-protein interaction (PPI) between LWD1 and the 219 biotinylated protein candidates using the AlphaPulldown package with pulldown mode (19). In addition to the suggested default settings, the max_recycles parameter was set to 15 to ensure convergence and quality of the model predictions (20). The TAIR10 representative gene models of LWD1 and the interacting candidate proteins were used for *in silico* PPI predictions. The interface predicted Template Modeling-score (ipTM-score) was generated from AlphaPulldown package to estimate PPI from the features of protein-protein interfaces. Only the scores from the top-ranked predicted interacting dimer were included in output. For quality assurance of complex predictions, the AlphaPulldown-provided values of inter-chain predicted alignment error (inter-PAE) were ≤ 10Å as displayed in the **Dataset S3**.

Bioluminescence measurement and data analyses

The F3 plants of *LWD1-TbID* in *lwd1lwd2 pCCA1:LUC2* and *nLWD1-TbID* in *lwd1lwd2 pCCA1:LUC2* were obtained by crossing *lwd1lwd2 pCCA1:LUC2* reporter line (2) with *LWD1-TbID lwd1lwd2* or *nLWD1-TbID lwd1lwd2* plants, respectively. The epigenetic and transcription factor mutants were crossed with *pCCA1:LUC2* reporter line (2). The homozygous F3 mutants harboring *pCCA1:LUC2* reporter were grown on 1/2 MS medium under 16-h light/8-h dark with ~80 µmole $m^{-2}s^{-1}$ of white light

illumination for 7-9 d. The individual seedlings were then transferred into black 96-well microplates containing 200 µL of 1/2 MS solid medium with 80 µL of 1/2 MS liquid medium, supplemented with 0.5 mM luciferin (PerkinElmer) to monitor the bioluminescence activities spanning a 1-d period under diurnal long-day conditions (16-h light/8-h dark), followed by 7-d continuous white light at 36 µmole $m^{-2}s^{-1}$ illumination.

Bioluminescence activities were measured in a light-proof chamber (Phenotron, HiPoint) with a SOPHIA 2048BX Large Format CCD Camera (Princeton Instruments) and LightField v6.10. The images of luciferase activity were recorded every 1 h for 7 d. Image processing and quantification were performed by image processing script (HiPoint). Period lengths and relative amplitude error (RAE) from 48 h to 120 h bioluminescence data under free-running conditions were estimated by fast Fourier transform-nonlinear least squares (FFT-NLLS) (21) using the Biological Rhythms Analysis Software System (BRASS) (available from http:// www.amillar.org) (22). The bioluminescence activity of *pCCA1:LUC2* in each transgenic line was normalized to its mean bioluminescence and plotted in Fig 1E, 6A, S8, S9C and S10C.

Hypocotyl length measurement

After stratification, the seeds germinated and seedlings grew in the dark or illuminated with 10 µmole $m^{-2}s^{-1}$ of continuous white light for 4 d. The hypocotyl length was measured by using ImageJ v1.47 (23). Student's t-test was used to evaluate differences in hypocotyl length between WT and mutant seedlings. Three biological replicates were performed.

Y2H assays

The full-length coding sequence (CDS) of *LWD1* was cloned into the pGBKT7 (Clontech) to generate the bait protein BD-LWD1, while the CDS of LWD1-interacting proteins, such as *PRR5*, *PRR7*, *TOC1*, *TPL*, *HDA19*, *TIC*, *AL2*, *HB23*, *TTA2*, *SEU*, *HDC1*, were cloned into the pGADT7 (Clontech) to generate the prey constructs (AD-LWD1-interacting proteins). The primer pairs used in the construction processes are listed in **Dataset S5**. Both constructs were co-transformed into the yeast strain AH109 using the lithium

acetate-mediated method (24). Transformed cells were selected on double-dropout medium (SD/-Leu (L)/-Trp (W)). To test for interactions, the resulting colonies were screened on high-stringent quadruple-dropout medium (SD/-Ade/-His/-L/-W). Yeasts co-transformed with empty vectors were used as negative controls. Plates were incubated at 28°C before colony growth was assessed.

In vitro pull-down assays

The coding sequences encoding maltose binding protein (MBP), GFP, LWD1, PRR5, PRR7, N-TOC1 (1-1296 bp), and C-TOC1 (1297-1854 bp) were amplified using gene-specific primers (**Dataset S5**) and cloned into the pET-28a vector for generating *pET28a-MBP-LWD1*, *pET28a-PRR5-GFP*, *pET28a-PRR7-GFP*, *pET28a-N-TOC1* (1-423 a.a.)-*GFP,* and *pET28a-C-TOC1* (424-618 a.a.)-*GFP* constructs. The resulting plasmids were transformed into *Escherichia coli* strain C43 (DE3). Protein expression was induced with 0.5 mM isopropyl-β-D-thiogalactoside (IPTG) at 16°C for 12-16 h. Cells were harvested by centrifugation (5,000 × g, 10 min) and lysed using BugBuster Master Mix reagent (Millipore) at 25°C for 10 min. Following lysis, the crude extract was cleared by centrifugation at 20,000 × g for 20 min at 4°C. For the pull-down, supernatants containing MBP-LWD1 or MBP (control) were incubated with anti-MBP magnetic beads (Sigma) for 1 h at 4°C with gentle rotation. The protein-immobilized beads were washed three times with 1X PBS (pH 7.4) containing 0.5% NP-40. Subsequently, the beads were incubated with total soluble proteins from *E. coli* expressing PRR5-GFP, PRR7-GFP, N-TOC1-GFP, or C-TOC1-GFP for 2 h at 4°C. After three washes with 1X PBS containing 0.5% NP-40 and three additional washes with 1X PBS to remove non-specific binding proteins, bound complexes were eluted in SDS-PAGE sample buffer at 90°C for 5 min. Proteins were resolved via SDS-PAGE and transferred to PVDF membranes for immunoblot analysis using anti-MBP (YH-80510, Yao-Hong Biotech) or anti-GFP (JL-8, Clontech) antisera and the HRP-conjugated anti-mouse antibody (W4021, Promega) as the secondary antibody.

RNA isolation and quantitative reverse transcription PCR (qRT-PCR) assays

Total RNA was extracted as previously described (25) with minor modifications. Total

RNA was isolated from 14-d-old LWD1-interacting protein mutants harvested at ZT15-16 under long-day (LD) conditions (16-h light/8-h dark) or 4-day-old seedlings harvested at ZT6 under LD conditions. cDNA was synthesized from DNase-treated RNA using SuperScript IV reverse transcriptase (Invitrogen) and oligo-dT primers. Real-time PCR was performed on a QuantStudio 12K Flex Real-Time PCR System (Applied Biosystems) using manufacturer-recommended protocols, primers designed via Primer Express (**Dataset S5**), and Power SYBR Green Master Mix (Applied Biosystems). Relative gene expression was determined using the comparative threshold cycle (CT) method, with *UBQ10* serving as the internal control. Mean values were calculated as $2^{-\Delta CT}$, where Δ CT = $CT_{(\textit{target gene})} - CT_{(\textit{UBQ10})}$. Data represent the mean of three technical replicates. Independent biological replicates were conducted with similar results.

Generation of constructs and transgenic lines

To generate the *pSEU:SEU-FLAG* construct, the *SEU* promoter (comprising 2,971 bp upstream of the translation start site), the *SEU* CDS, and a *3×FLAG* DNA fragment were amplified using gene-specific primers (**Dataset S5**). These fragments were assembled into *Pvu*II/*Sac*I-digested *pCAMBIA1390-Gen* (a modified *pCAMBIA1390* carrying a gentamycin resistance gene) via homologous recombination using the UltraFast Recombinase Mix (Tools), following the manufacturer's instructions. The resulting *pSEU:SEU-FLAG* plasmid was then transformed into the *seu-1 pCCA1:LUC2* transgenic line using the floral dip method (7).

To generate the *p35S:HB23* construct, the *35S* promoter, *HB23* CDS, and a *3×HA* DNA fragments were amplified using gene-specific primers (**Dataset S5**) and cloned into *Pvu*II/*Sac*I-digested *pCAMBIA1390-Gen*. To generate the *p35S:HB23-VP16* and *p35S:HB23-SRDX* constructs, sequences encoding the VP16 transcriptional activation domain or the SRDX transcriptional repression motif were amplified from *MYB16-VP16* or *MYB16-SRDX* plasmids (26), respectively. These fragments were then cloned into the *p35S:HB23* vector linearized with *SacI* and treated with Calf Intestinal Alkaline Phosphatase (CIP; NEB). All three constructs were independently transformed into the *pCCA1:LUC2* transgenic line via the floral dip method (7).

To generate the estrogen inducible expression construct *pER8:LWD1-EYFP-HA*, the

gene-specific primers (**Dataset S5**) were used to amplify the *LWD1* coding region and the *EYFP-3HA* fragment. The two DNA fragments were assembled with *Xho*I/*Spe*I linearized *pER8* backbone with a bialaphos resistance gene (27) using NEBuilder HiFi DNA assembly master mix (NEB) to obtain *pER8:LWD1-EYFP-HA*. The *pER8:LWD1-EYFP-HA* was transformed separately into the reporter lines *pCCA1:LUC2* or *lwd1lwd2 pCCA1:LUC2* (2) using the floral dip method (7).

Chromatin Immunoprecipitation (ChIP)-qPCR

ChIP was performed according to a previous report (28) with modifications. Two-week-old seedlings were harvested at ZT16 under long-day conditions and crosslinked with 1% formaldehyde for 20 min at room temperature with gentle vacuum. The seedlings were then quenched by 125 mM glycine for 5 min, and then washed three times with ice-cold water. The blot-dried seedlings were ground with liquid nitrogen and suspended in extraction buffer I [10 mM Tris-HCl pH 7.4, 0.4 M sucrose, 1 mM PMSF, 50 µM MG132, and 50x diluted (v/v) protease inhibitor cocktail (Roche)]. The crude extracts were sequentially filtered through 70 µm and 40 µm nylon meshes, after which the filtrates were centrifuged at 3,000 × g at 4 °C for 10 min to pellet the nuclei. The pellets were resuspended in extraction buffer II [10 mM Tris-HCl pH 7.4, 0.25 M sucrose, 10 mM MgCl2, 1% Triton X-100, 1 mM PMSF, 50 µM MG132, and 50x diluted (v/v) protease inhibitor cocktail (Roche)] and centrifuged at 12,000 x g at 4 °C for 10 min. After removing the supernatant, the pellets were thoroughly resuspended in extraction buffer III [10 mM Tris-HCl pH 7.4, 1.7 M sucrose, 2 mM MgCl2, 0.15% Triton X-100, 1 mM PMSF, 50 µM MG132, and 50x diluted (v/v) protease inhibitor cocktail (Roche)]. The resuspended pellets were carefully layered onto the same volume of extraction buffer III and centrifuged at 16,000 × g at 4 °C for 1 h. The isolated nuclei pellets were thoroughly suspended in cold nuclei lysis buffer [50 mM Tris-HCl pH 7.4, 100 mM NaCl, 2 mM EDTA, 0.5% Sarkosyl, 1 mM PMSF, 50 µM MG132, and 50x diluted (v/v) protease inhibitor cocktail (Roche)] at 4 °C, and the samples were gently rotated for 20 min to efficiently release chromatin.

Chromatin samples were sheared to an average DNA fragment size of 1-1.5 kb using a Bioruptor UCD-200 (Diagenode). Sonication was performed for 15 cycles (30 sec ON/1 min OFF) on high-strength mode at 4 °C. Debris was removed by centrifugation at

16,000 × g for 10 min at 4°C. One percent of the clarified supernatants were set aside as the input control.

The remaining chromatin lysates were individually diluted with 4 volumes of ChIP dilution buffer [50 mM Tris-HCl pH 7.4, 100 mM NaCl, 2 mM EDTA, 1.25% Triton X-100, 1 mM PMSF, 50 μM MG132, and 50x diluted (v/v) protease inhibitor cocktail (Roche)], and then incubated with 50 μL anti-FLAG magnetic beads (M8823, Sigma) at 4°C with gentle rotation for 4 h. The beads were sequentially washed with 1 mL of the following buffers: 2 times of low salt wash buffer [50 mM Tris-HCl pH 7.4, 150 mM NaCl, 2 mM EDTA, 0.5% Triton X-100], 2 times of high salt wash buffer [50 mM Tris-HCl pH 7.4, 500 mM NaCl, 2 mM EDTA, 0.5% Triton X-100], and 3 times of Tris-EDTA Buffer [50 mM Tris-HCl pH 7.4, 50 mM NaCl, 2 mM EDTA].

To reverse the formaldehyde cross-links, both the input and the immunoprecipitated samples were mixed with 100 μL ChIP elution buffer [50 mM Tris-HCl pH 8.0, 1% SDS, 10 mM EDTA]. Proteinase K (Invitrogen) was added to a final concentration of 0.2 mg/mL and incubated at 65°C overnight. The eluted DNA was transferred to a fresh tube. DNA was subsequently purified using MinElute PCR Purification Kit (QIAGEN) and eluted in 100 μL nuclease-free water.

Quantitative PCR was performed in triplicate using SYBR Green Master Mix (Applied Biosystems) on an Applied Biosystems QuantStudio 5 as described (9). The primers utilized to amplify the target regions were listed in **Dataset S5**. *UBC21* was used as negative control. ChIP-qPCR data were normalized to that of the input sample.

Dual Luciferase Assays

To assess the function of LWD1 in PRR-TPL complex, protoplasts were isolated from *pER8:LWD1-EYFP-HA lwd1lwd2 pCCA1:LUC2* seedlings and transfected with plasmids indicated in **Fig. 6B** at ZT11 as described (10, 11) with *p35S:GFP* as a negative control and *pUbi10:Renilla luciferase* (R-Luc) as an internal control for transfection efficiency normalization. The *LWD1-EYFP* expression was induced at ZT12 by 10 μM β-estradiol or with DMSO treatment as a negative control. Soluble protein samples were collected at ZT18 and diluted for a dual luciferase assay according to the manufacturer's instructions (Promega, E1910).

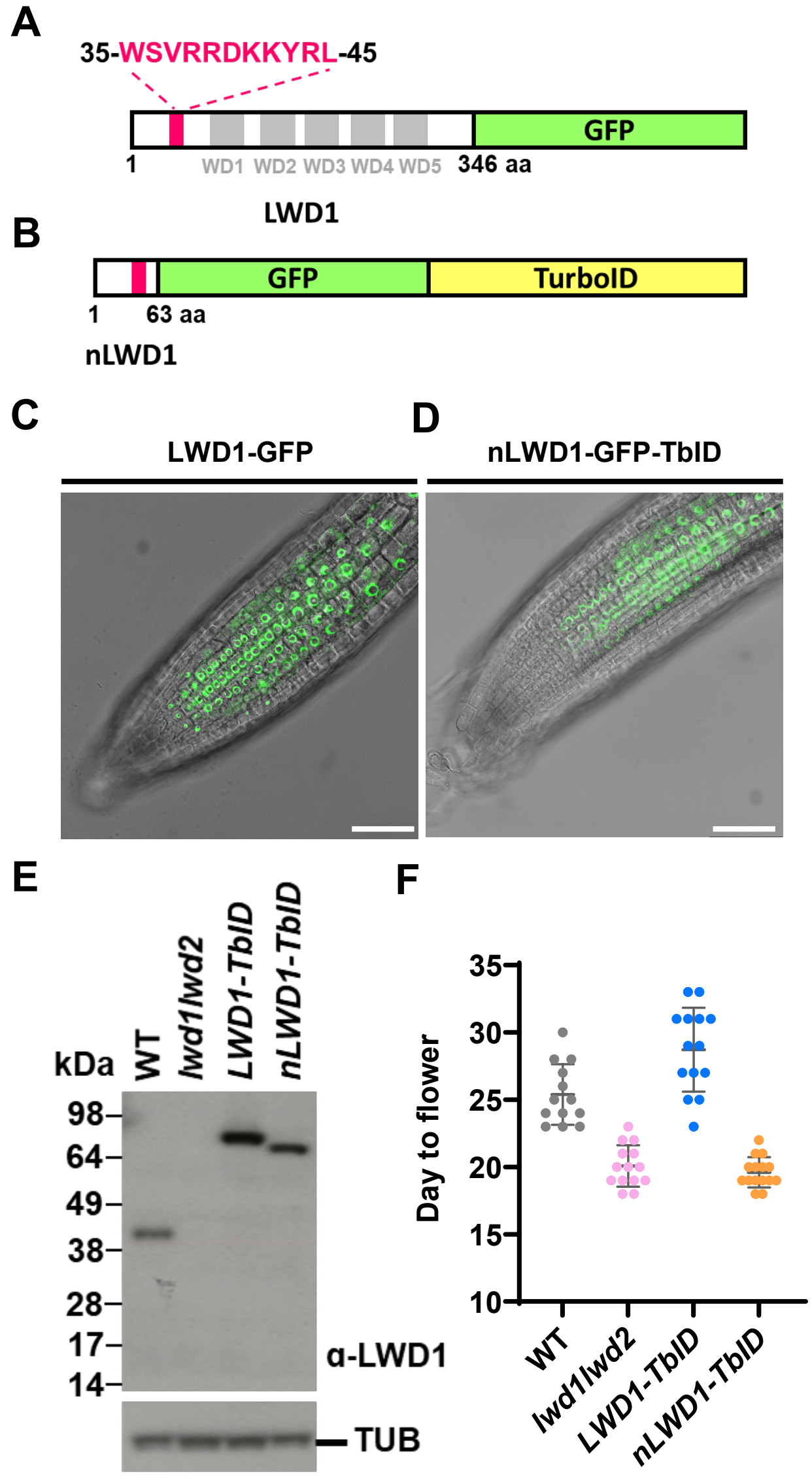

Fig. S1. Characterizations of *LWD1-TbID* and *nLWD1-TbID* seedlings.

(A) LWD1-GFP protein structure. Potential nuclear localization signal (NLS) of LWD1 located at amino acid 35 to 45 was predicted by using the machine learning-based identification of nucleus signal peptide server (http://www.csbio.sjtu.edu.cn/ bioinf/INSP/) with a score of 0.928. Five Tryptophan-Aspartate (WD) domains of LWD1 were also marked. **(B)** nLWD1-GFP-TurboID protein structure. **(C and D)** LWD1-GFP (**C**) and nLWD1-GFP-TbID (nLWD1-TbID) (**D**) driven by native *LWD1* promoter localize in nucleus in root tip of 5-d-old transgenic seedlings. Scale bar, 50 µm. **(E)** The expression levels of LWD1, LWD1-TbID and nLWD1-TbID proteins. The crude protein extracts were extracted from 14-d-old WT, *lwd1lwd2*, *LWD1-TbID*, and *nLWD1-TbID* seedlings. Immunoblot analysis was performed using LWD1-specific antiserum and tubulin antiserum for loading control. **(F)** Flowering time of WT, *lwd1lwd2*, *LWD1-TbID*, and *nLWD1-TbID* were recorded when the primary inflorescence reached ~3 cm. Data are means with standard deviation (s.d.) of ≥ 9 plants. Three biological replicates showed similar results.

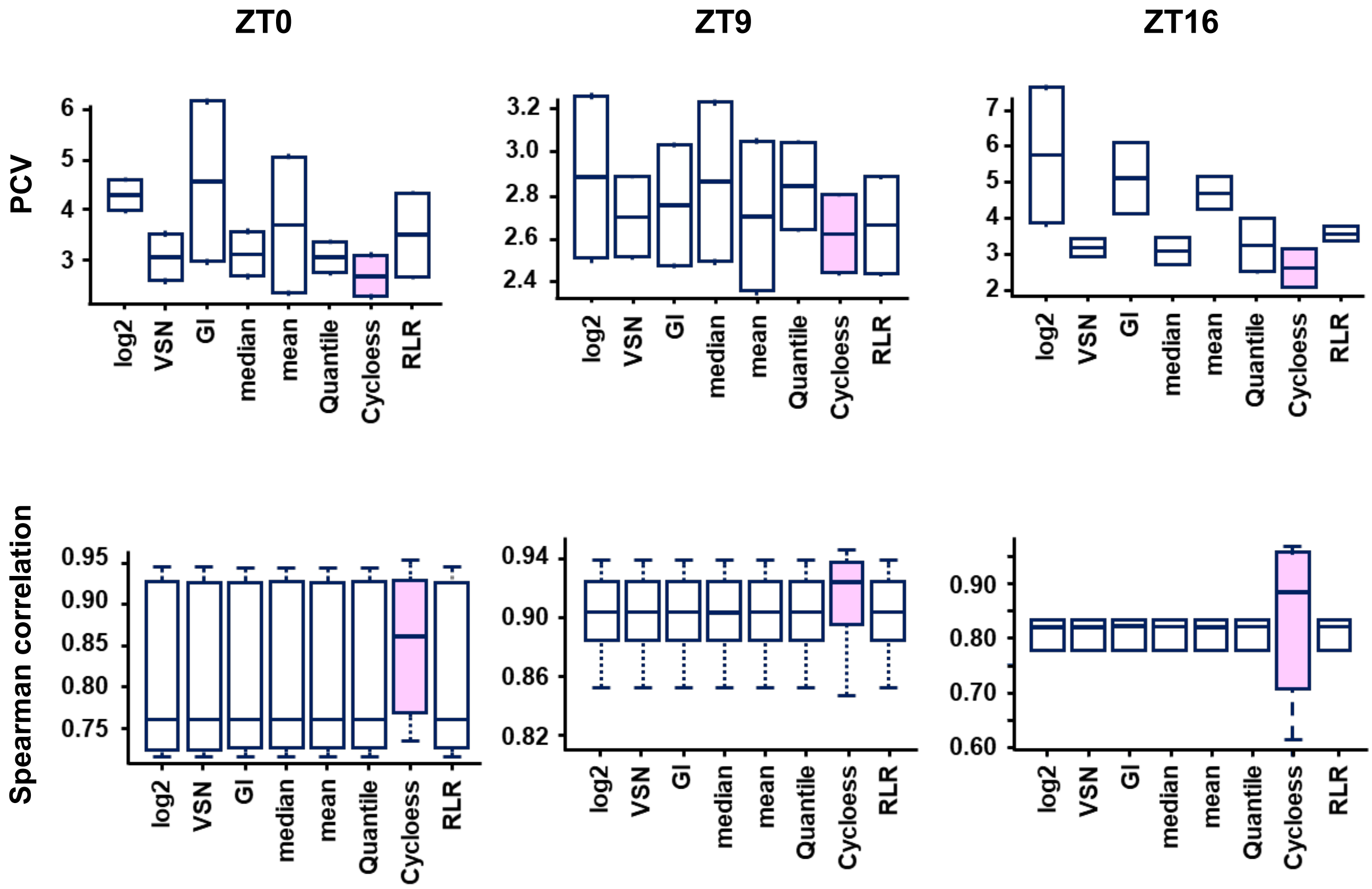

Fig. S2. CycLoess normalization showed minimal variance and superior correlation for LFQ results.
The NormalyzerDE online tool was used to compare 8 data normalization methods, including log2, variance stabilization normalization (VSN), global intensity (GI), median intensity, mean intensity, quantile, cyclic locally estimated scatterplot smoothing (CycLoess), and robust linear regression (RLR). The pairwise variances and correlations for triplicated ZT0, ZT9, and ZT16 samples were calculated. Results indicated that CycLoess normalization had the lowest pooled coefficient of variation (PCV) and the highest Spearman correlation for samples from all 3 ZTs.

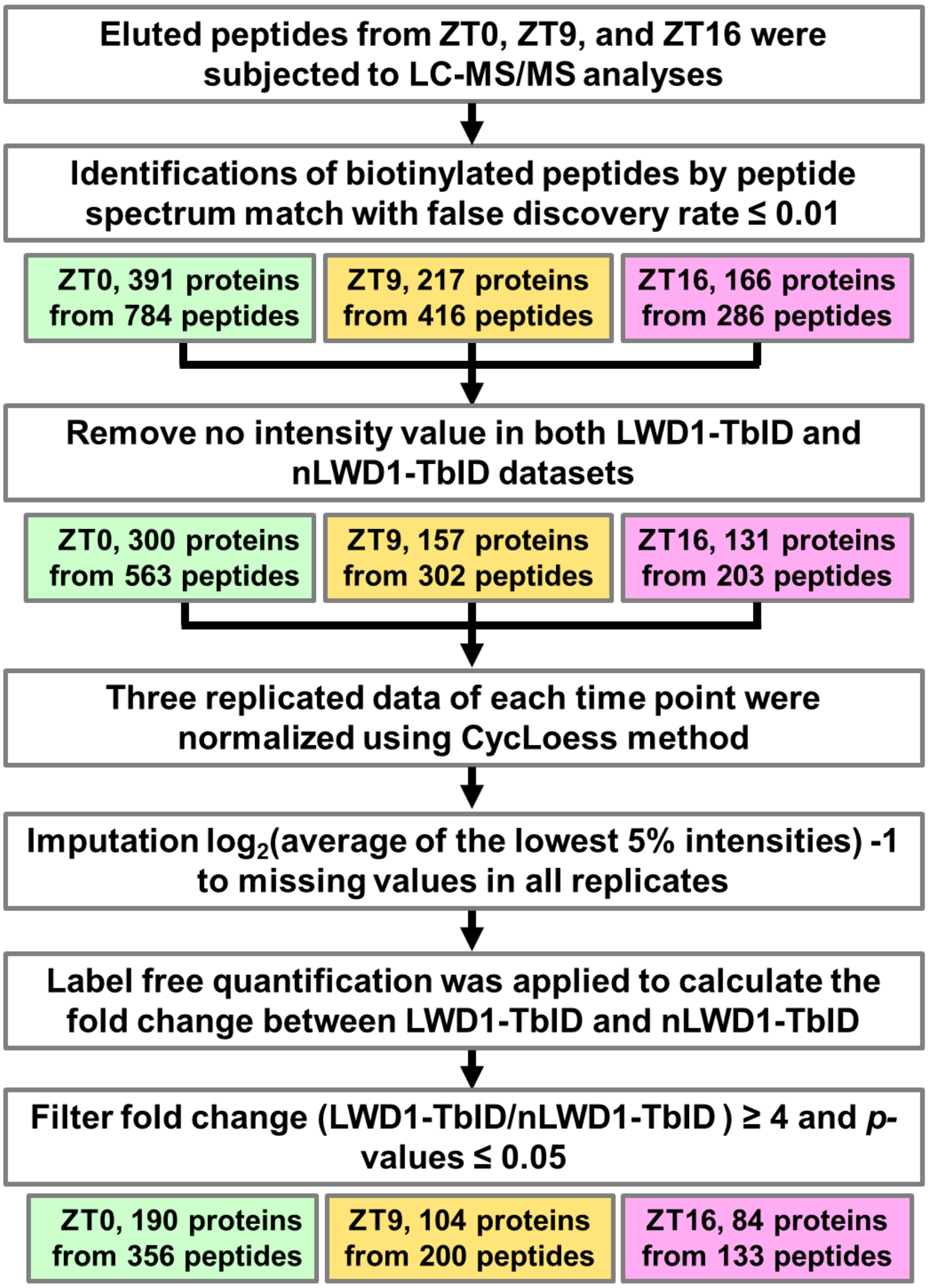

Fig. S3. A flowchart of experimental and data processing procedures.
Experimental steps and filtering criteria used in this study for identification of LWD1 interacting protein candidates with a label-free quantification approach.

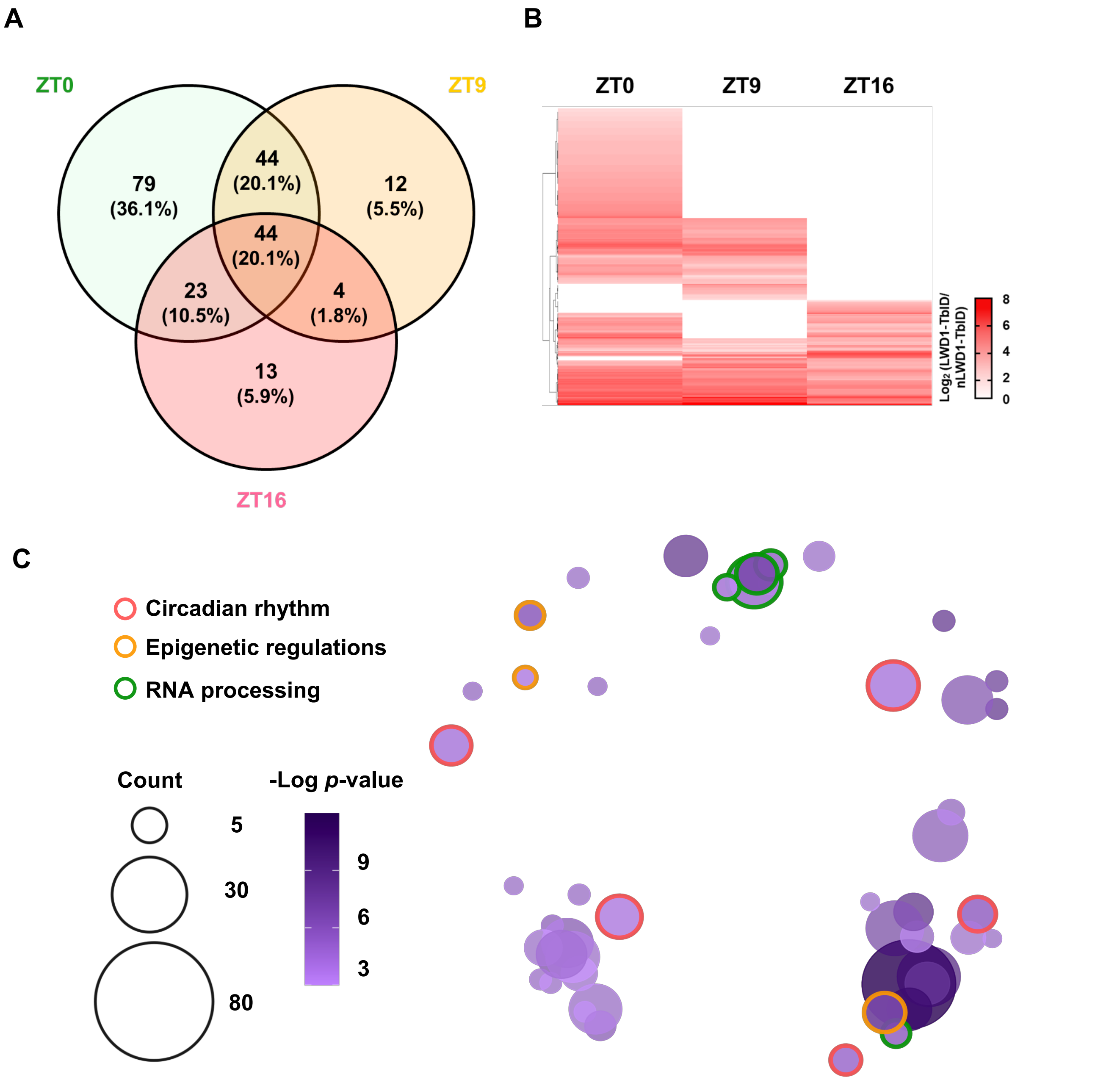

Fig. S4. Representation of LWD1-interacting proteins at 3 ZTs and their functional enrichment in circadian rhythm, epigenetic regulation, and RNA processing.

(A) Venn diagram showing more than 50% of the enriched biotinylated proteins were identified in at least 2 out of the 3 ZT samples. **(B)** A heat map showing the fold changes of the 219 candidate LWD1-interacting proteins at 3 ZTs. Color strip on the right indicates the $\log_2$ ratios between LWD1-TbID and nLWD1-TbID. **(C)** Gene ontology (GO) enrichment analysis was applied for the 219 LWD1-interacting candidates. The biological process of redundant GO-terms in the same clade were removed. Sphere sizes are proportional to the number of gene counts. Each sphere was color-coded according to their enrichment probability ($-\log_{10}$(*p*-value)). GO terms related to circadian rhythm, epigenetic regulation, and RNA processing were highlighted in red, orange, and green, respectively. The illustration was prepared by using the REVIGO software (http://revigo.irb.hr/).

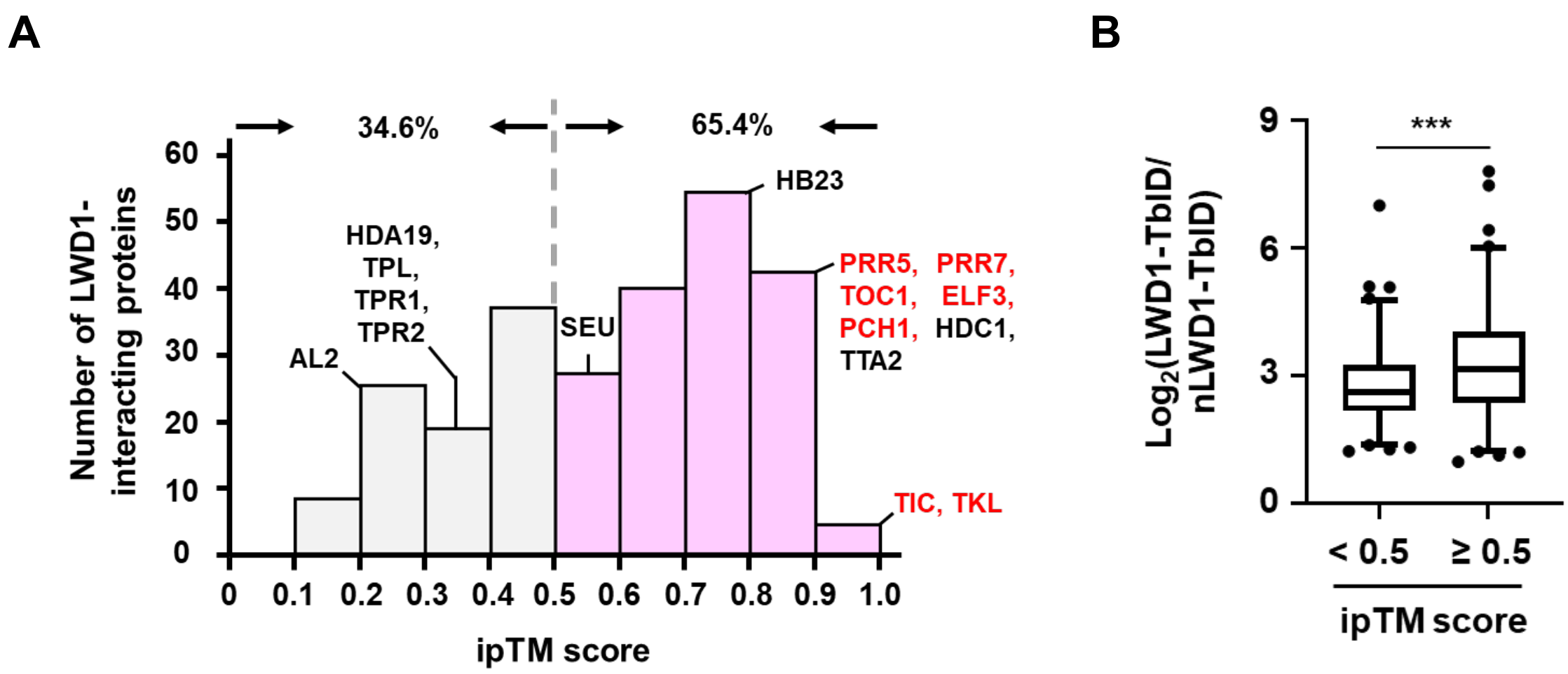

Fig. S5. *In silico* prediction of interactions between LWD1 and the 219 candidate interacting proteins.
(A) Distribution of interface predicted Template Modeling-score (ipTM-score) between LWD1 and the 219 candidate interacting proteins. The ipTM score was calculated using AlphaFold-Multimer with pull-down mode and the cut-off of 0.5 was considered a confident prediction. Key circadian components and epigenetic regulators are marked in red and black, respectively. **(B)** Box plot of biotinylated protein enrichment level [Log_2(LWD1-TbID/nLWD1-TbID)] for those with low ipTM-score (n=73) and high ipTM-score (n=146). *p*-value <0.0001 (***) was calculated by using two-tailed Wilcoxon signed rank test.

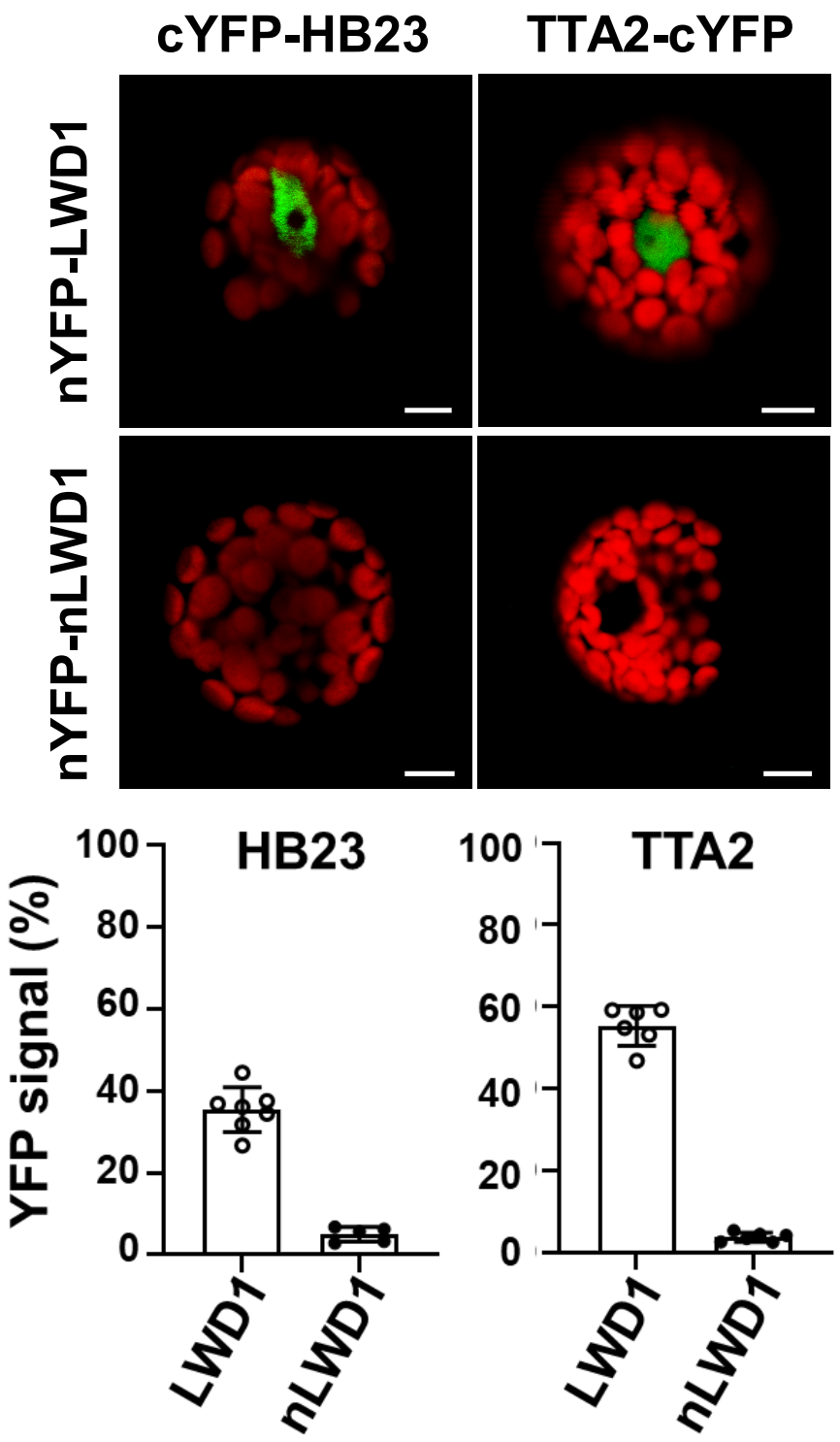

Fig. S6. Interactions between LWD1 and transcriptional factors validated by BiFC assay.
cYFP-fused HB23 or TTA2 were co-expressed with nYFP-fused LWD1 or nLWD1 in Arabidopsis protoplasts. Yellow fluorescent protein (YFP) signals (in green) indicated interactions between LWD1 and its candidate partners. Representative images are shown (top panel). The percentage of protoplasts displaying nuclear YFP signals in experimental (LWD1) and control (nLWD1) was calculated (n ≥ 200 protoplasts in each of the three biological replicates).

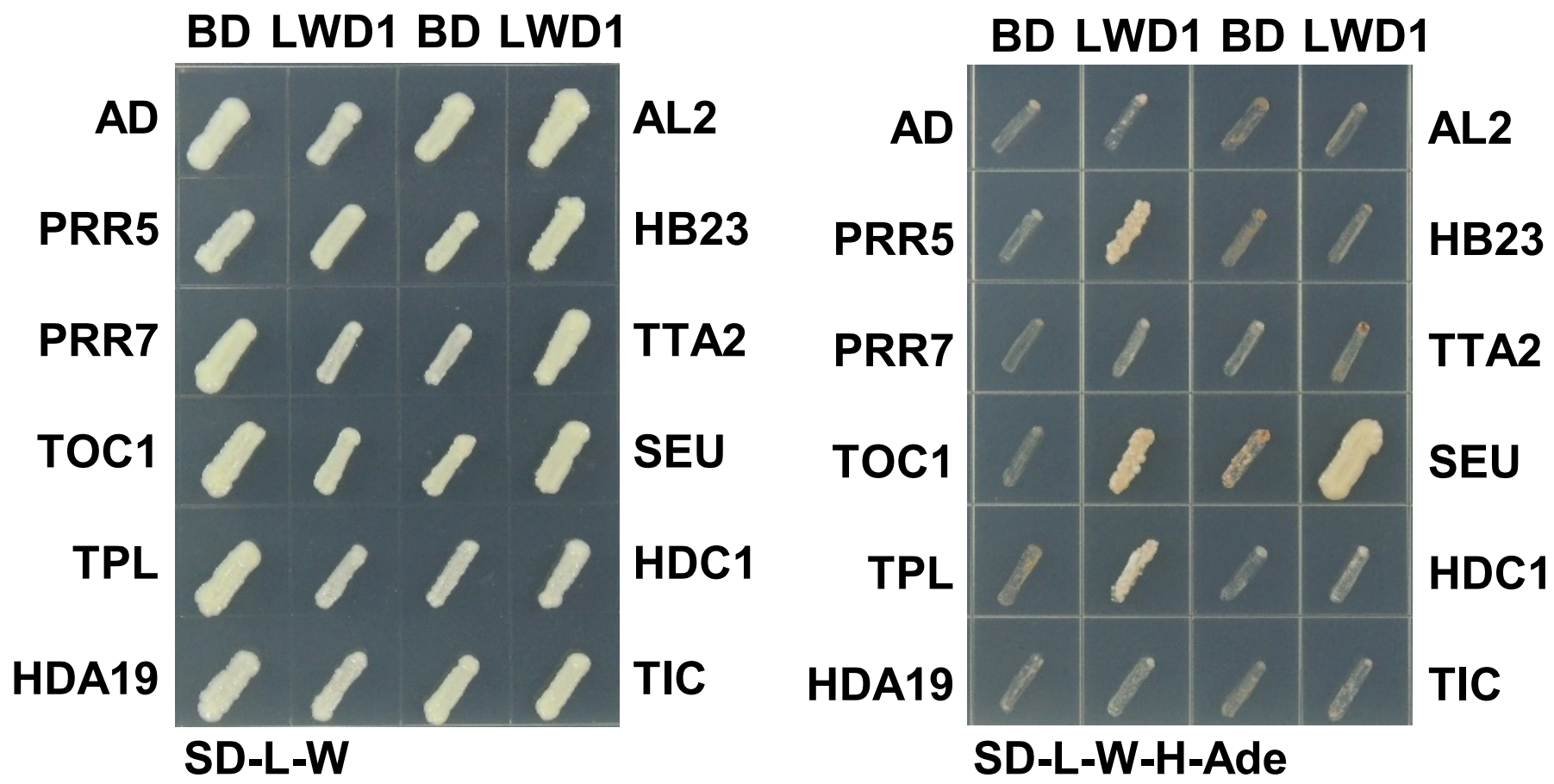

Fig. S7. LWD1 interacts with PRR5, TOC1, TPL and SEU proteins by yeast two-hybrid assays.
Yeasts co-transformed with the indicated BD-LWD1 (bait) and AD-tagged candidate proteins (preys) were grown on non-selective media (left: SD/-Leu (L)/-Trp (W)) to confirm successful transformation and cell viability, and on selective media (right: SD/-L/-W/-His(H)/-Ade) to examine the pairwise protein-protein interactions between bait and prey proteins. Co-transformation of the BD domain only with AD-tagged proteins served as negative controls. Four biological replicates showed similar results.

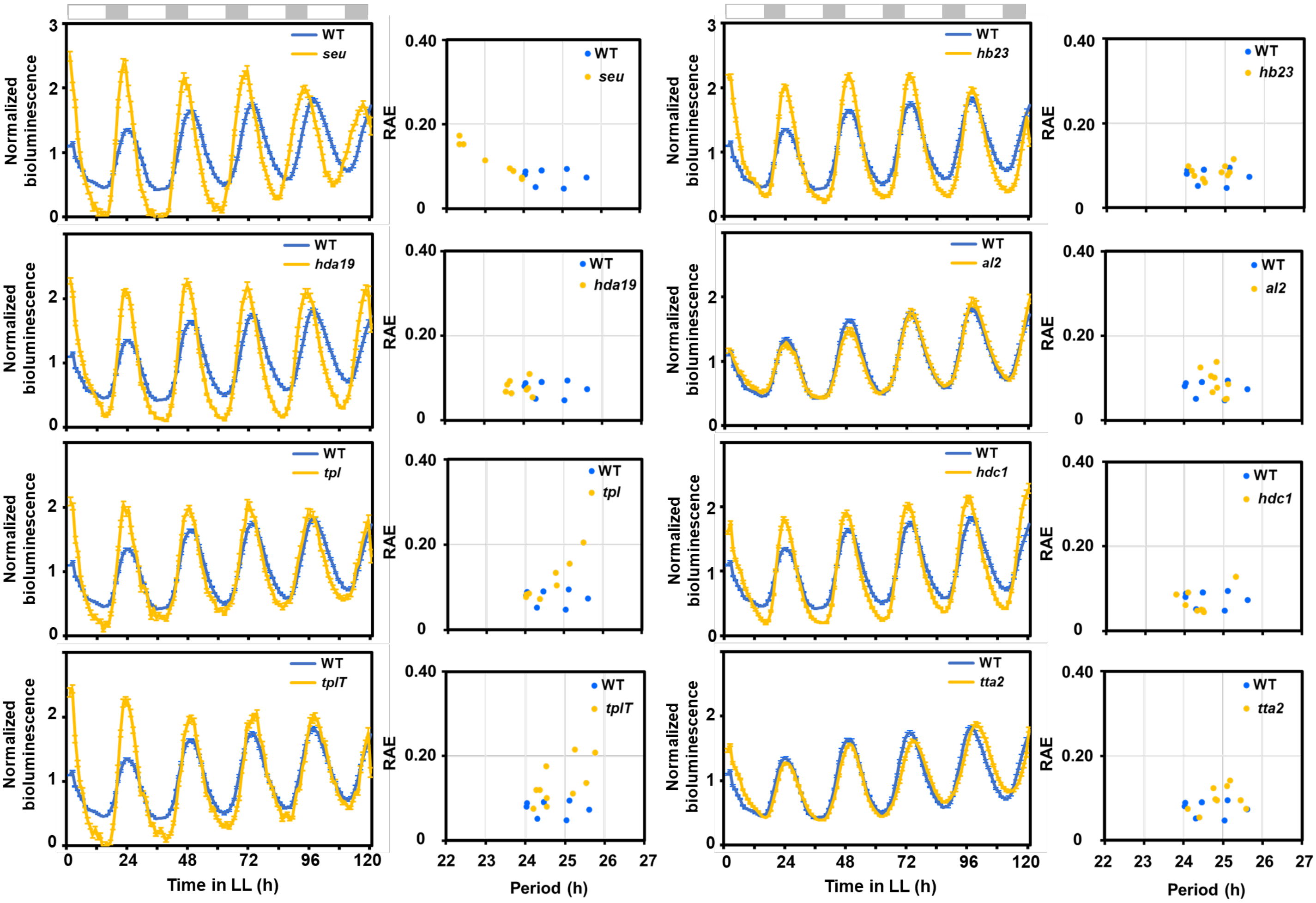

Fig. S8. Circadian behaviors of mutants defective in LWD1-interacting proteins.

Seedlings of *seu*, *hda19*, *tpl*, *tplT*, *hb23*, *al2*, *hdc1* and *tta2* mutants carrying *pCCA1-LUC2* reporter gene were entrained under a 16-h light and 8-h dark cycle for 7-9 days and transferred to continuous white light (LL, 36 µmol $m^{-2}s^{-1}$) to monitor bioluminescence. The period length and relative amplitude error (RAE) (right side) were determined by FFT-NLLS analysis with 95% confidence by using data from LL48 to LL120. At least 8 seedlings per genotype were used. The measurements were replicated three times with consistent results. White and gray bars denote the light and subjective darkness, respectively.

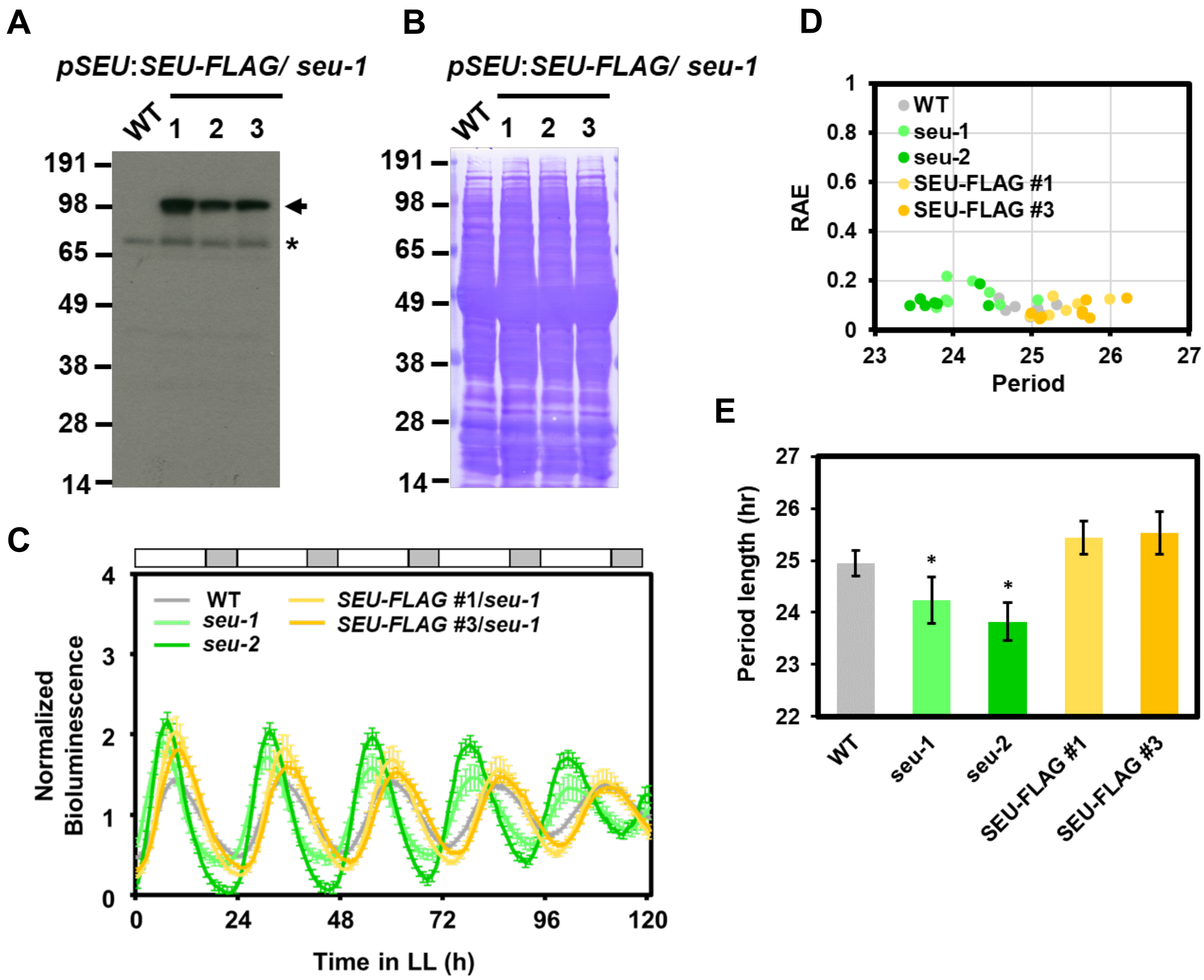

Fig. S9. The short-period phenotype of *seu* mutants can be rescued in the *seu-1 SEU-FLAG* complementation lines.

(A) The expression of SEU-FLAG protein in three independent *seu-1 pSEU:SEU-FLAG* complementation lines. Total proteins were extracted from 7-d-old seedlings under long-day conditions (16-h light/8-h dark). Anti-FLAG antiserum was used in the Immunoblot analysis. An arrow marked the SEU-FLAG protein, and an asterisk indicated a non-specific band. **(B)** Coomassie Brilliant Blue-stained blot as a loading control. **(C)** The normalized promoter activities of *pCCA1:LUC2* in WT (grey), *seu-1* (light green), *seu-2* (dark green), and two *SEU-FLAG* complementation lines (yellow and orange) were measured by bioluminescence assays. Data are means with standard errors (s.e.) of ≥ 8 seedlings. White and gray bars denote the light and subjective darkness, respectively. **(D)** A scatter plot representing the rhythmicity and period length of individual seedlings. The period length and relative amplitude error (RAE) of WT, *seu-1*, *seu-2*, and two *seu-1 pSEU:SEU-FLAG* lines carrying the *pCCA1:LUC2* reporter gene were calculated by the FFT-NLLS analysis from LL24 to LL120. **(E)** A bar graph showing the circadian period lengths of WT, *seu-1*, *seu-2*, two *seu-1 pSEU-FLAG* lines. Data are means with standard deviations (s.d.) of ≥ 8 seedlings. Three independent experiments were performed with similar results for **(D, E)**. Significant difference relative to WT was assessed using Student's t-test * indicates p-value ≤ 0.001.

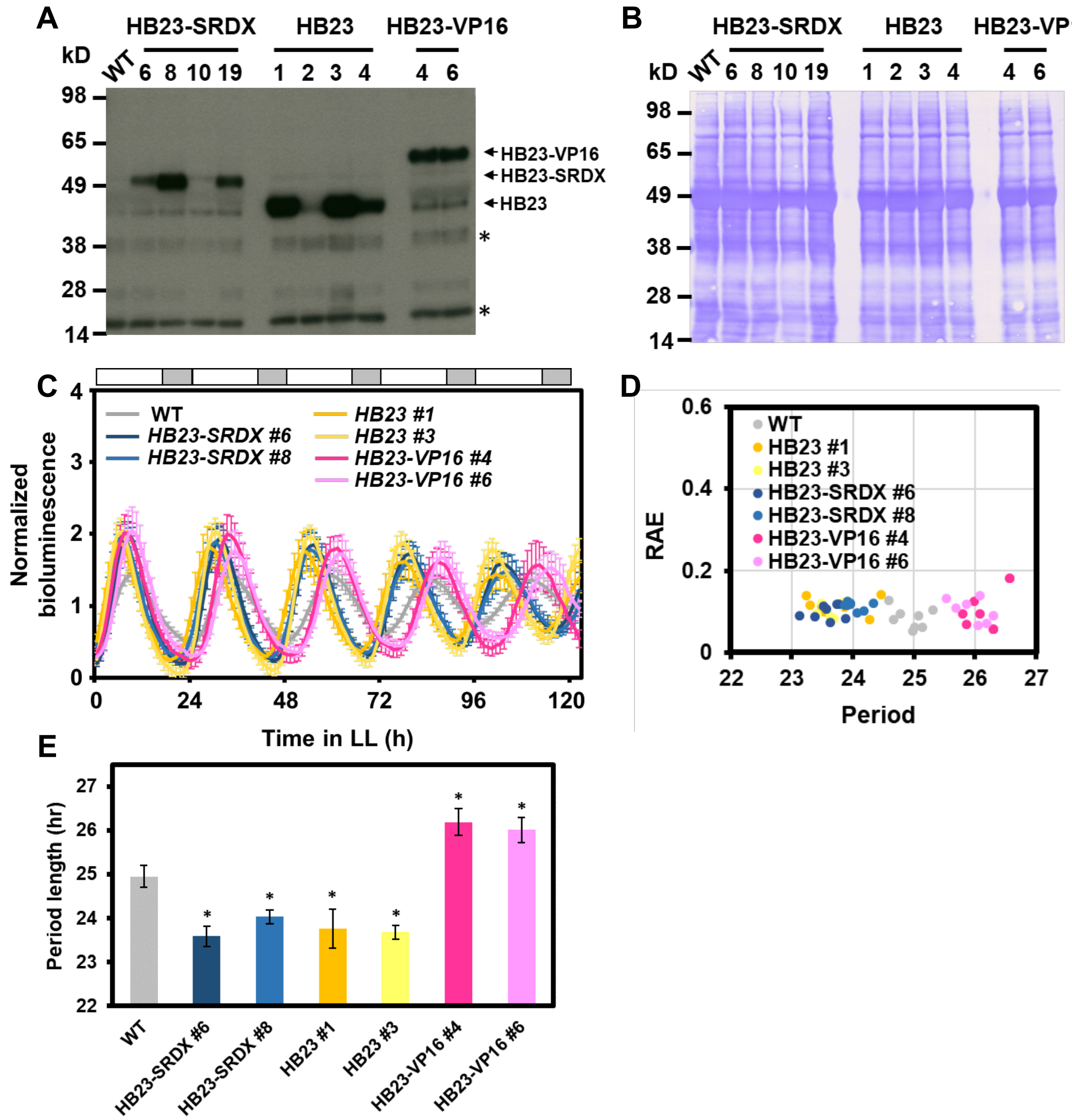

Fig. S10. HB23 is a novel regulator of Arabidopsis circadian clock.
(A, B) The expression of HB23-SRDX, HB23, and HB23-VP16 in independent transgenic lines. Total proteins were extracted from 7-d-old seedlings under long-day conditions (16-h light/8-h dark). Anti-FLAG antiserum was used in the Immunoblot analysis. Arrows marked the HB23-SRDX, HB23, and HB23-VP16 proteins, and an asterisk indicated a non-specific band. **(B)** Coomassie Brilliant Blue-stained blot as a loading control. **(C)** The normalized promoter activities of *pCCA1:LUC2* in WT (grey), *HB23-SRDX* #6 (dark blue), *HB23-SRDX* #8 (light blue), *HB23* #1 (orange), *HB23* #3 (yellow), *HB23-VP16* #4 (magenta), and *HB23-VP16* #6 (pink) were measured by bioluminescence assays. Data are means with standard errors (s.e.) of ≥ 8 seedlings. White and gray bars denote the light and subjective darkness, respectively. **(D)** A scatter plot representing the rhythmicity and period length of individual seedlings. The period length and RAE of WT, *HB23-SRDX* #6, *HB23-SRDX* #8, *HB23* #1, *HB23* #3, *HB23-VP16* #4, and *HB23-VP16* #6 seedlings carrying *pCCA1:LUC2* reporter gene were calculated by the FFT-NLLS analysis from LL24 to LL120. **(E)** A bar graph showing the circadian period lengths of WT, *HB23-SRDX*, *HB23*, *HB23-VP16* transgenic lines carrying *pCCA1:LUC2* reporter gene. Data are means with standard deviations (s.d.) of ≥ 8 seedlings. Three independent experiments were performed with similar results for **(C, D, E)**. Significant difference relative to WT was assessed using Student's t-test * indicates p-value ≤ 0.001.

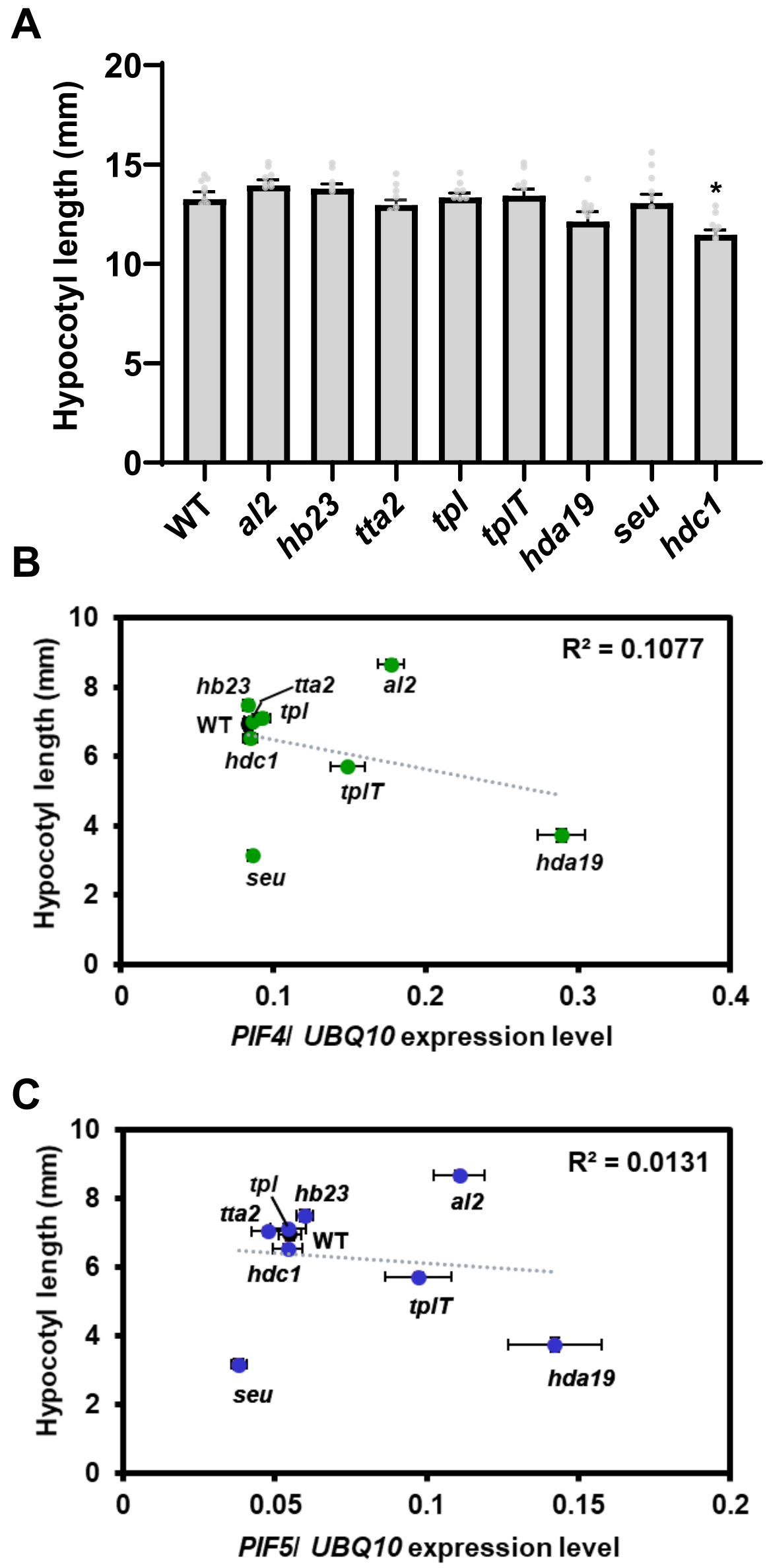

Fig. S11. Correlation analyses of *PIF4/PIF5* expression with hypocotyl lengths of mutants defective in LWD1-interacting proteins.

(A) The Bar graph showing hypocotyl lengths of 4-d-old dark-grown WT and mutant seedlings (n ≥ 10). Significant difference relative to WT was assessed using one-way ANOVA with Dunnett's multiple comparison test. * indicates p-value ≤ 0.05. Three biological experiments were performed with similar results. **(B, C)** Scatter plots showing the relationship between the expression levels of *PIF4* and *PIF5,* and the hypocotyl lengths of mutants defective in LWD1-interacting proteins. Total RNAs were extracted from 4-d-old WT and the mutants grown under long-day condition (16-h light/8-h dark) at ZT6. Gene expression levels were measured by qRT-PCR and normalized to *UBQ10* as an internal control. Each data point represents the mean of four biological replicates, with error bars indicating standard error (s.e.). The hypocotyl length data used were from those shown in Fig. 4B. WT is indicated by a filled black circle. Linear regression lines and the coefficient of determination (R^2) were also marked.

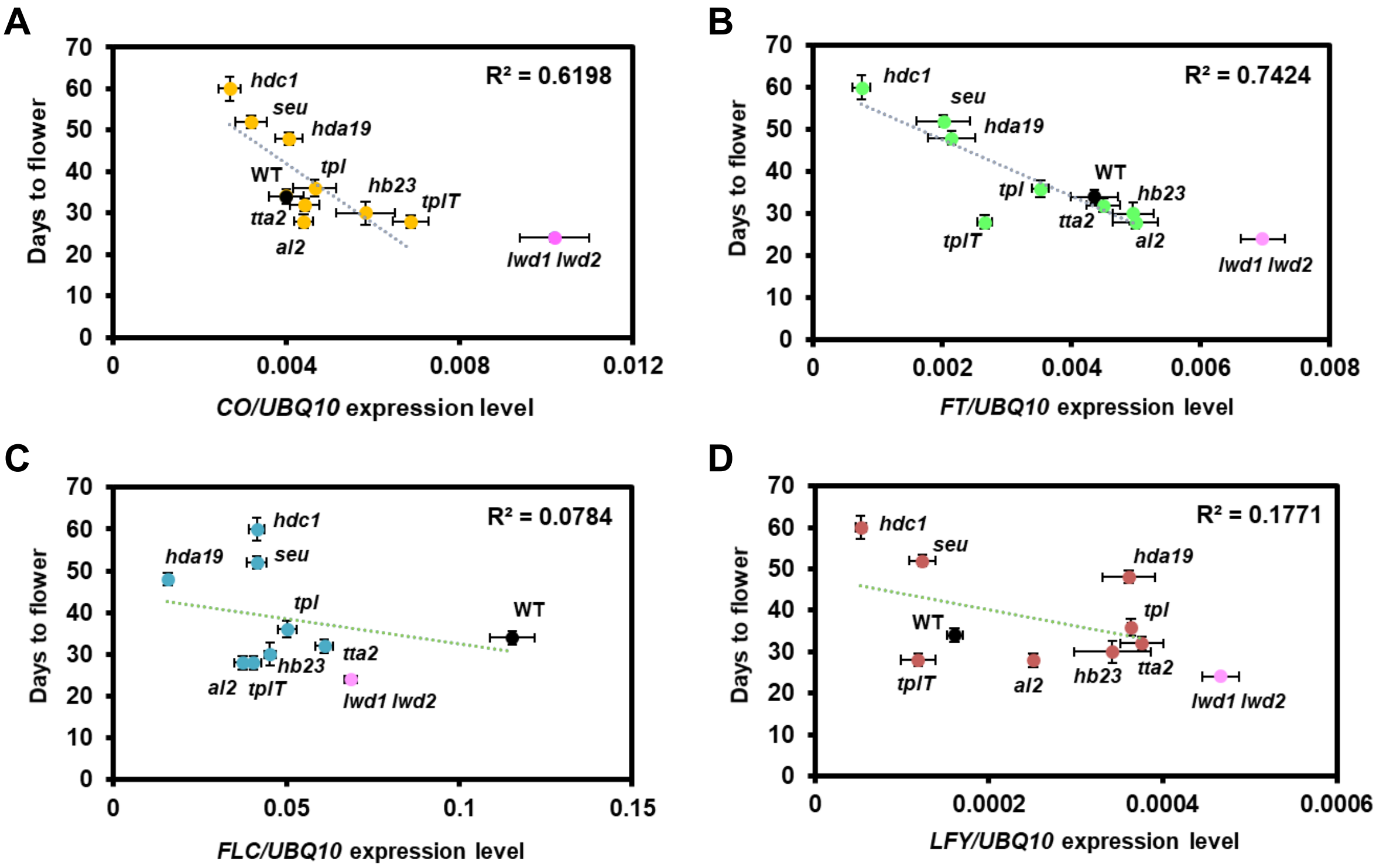

Fig. S12. Correlation analyses of *CO*, *FT, FLC,* and *LHY* expression with flowering time in mutants defective in LWD1-interacting proteins.

(A-D) Scatter plots showing the relationship between the expression levels of key flowering regulator genes, *CO*, *FT, FLC,* and *LHY*, and the number of days to flowering in mutants defective in LWD1-interacting proteins. Total RNAs were extracted from 4-d-old WT and mutants grown under long-day condition (16-h light/8-h dark) at ZT15-16. Gene expression levels were measured by qRT-PCR and normalized to *UBQ10* as an internal control. Each data point represents the mean of four biological replicates, with error bars indicating standard error (s.e.). The flowering time data used were from those shown in Fig. 4E. WT and *lwd1 lwd2* are indicated by filled black and pink circles, respectively. Linear regression lines and the coefficient of determination (R^2) were also marked.

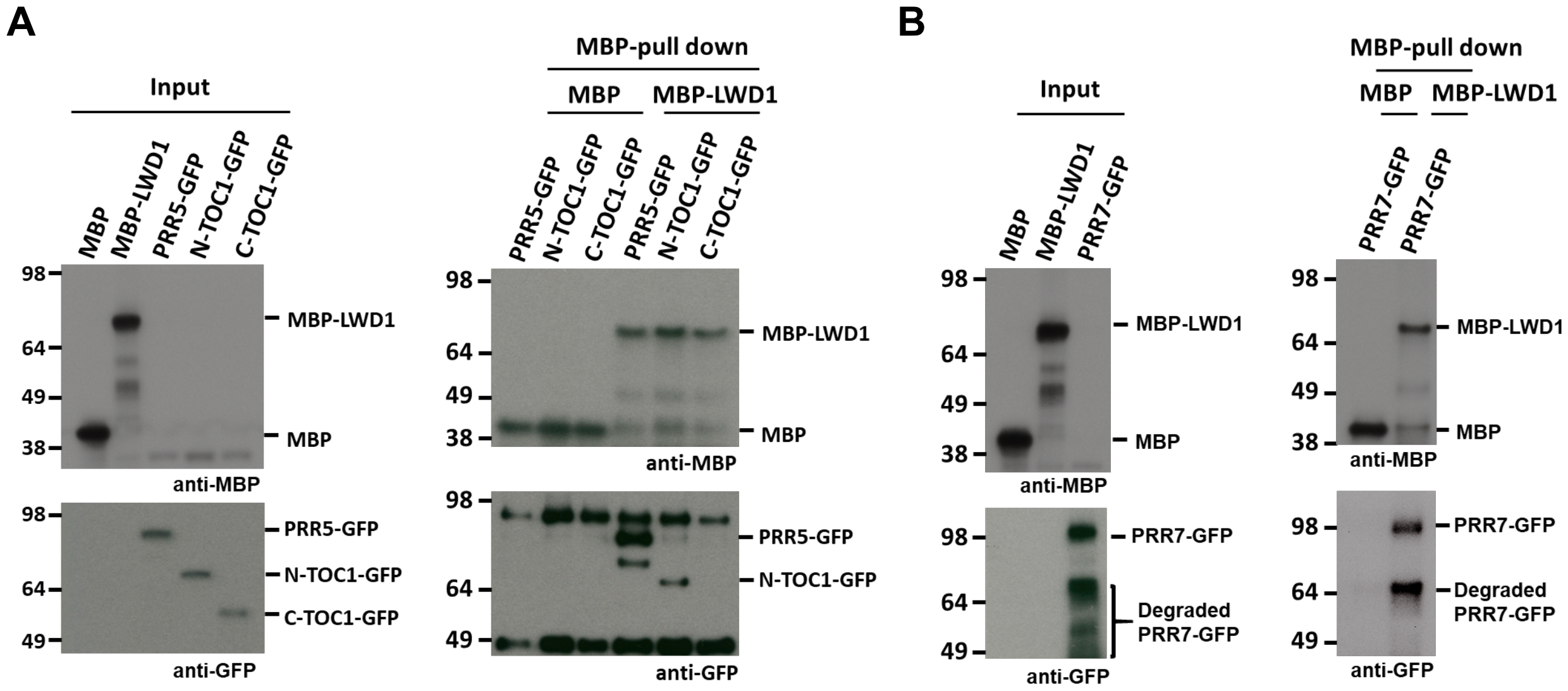

Fig. S13. Validating the interaction between LWD1 and PRR5, PRR7 and TOC1 proteins by *in vitro* pull-down assays.

In vitro pull-down assays showing LWD1 interacts with PRR5 and N-terminal TOC1 proteins **(A)** and PRR7 **(B)**. Protein extracts from *E. coli* expressing bait proteins (MBP-LWD1 and MBP as a negative control) and prey proteins (PRR5-GFP, PRR7-GFP, N-TOC1-GFP, or C-TOC1-GFP were used for *in vitro* pull-down assays as described in Methods. Left and right panels showed immunoblot analyses of the input proteins and the pull-down fractions, respectively. Upper and bottom panels were probed with anti-MBP and anti-GFP antisera, respectively. Molecular weights (kDa) and proteins are labeled. Three independent experiments were performed with similar results.

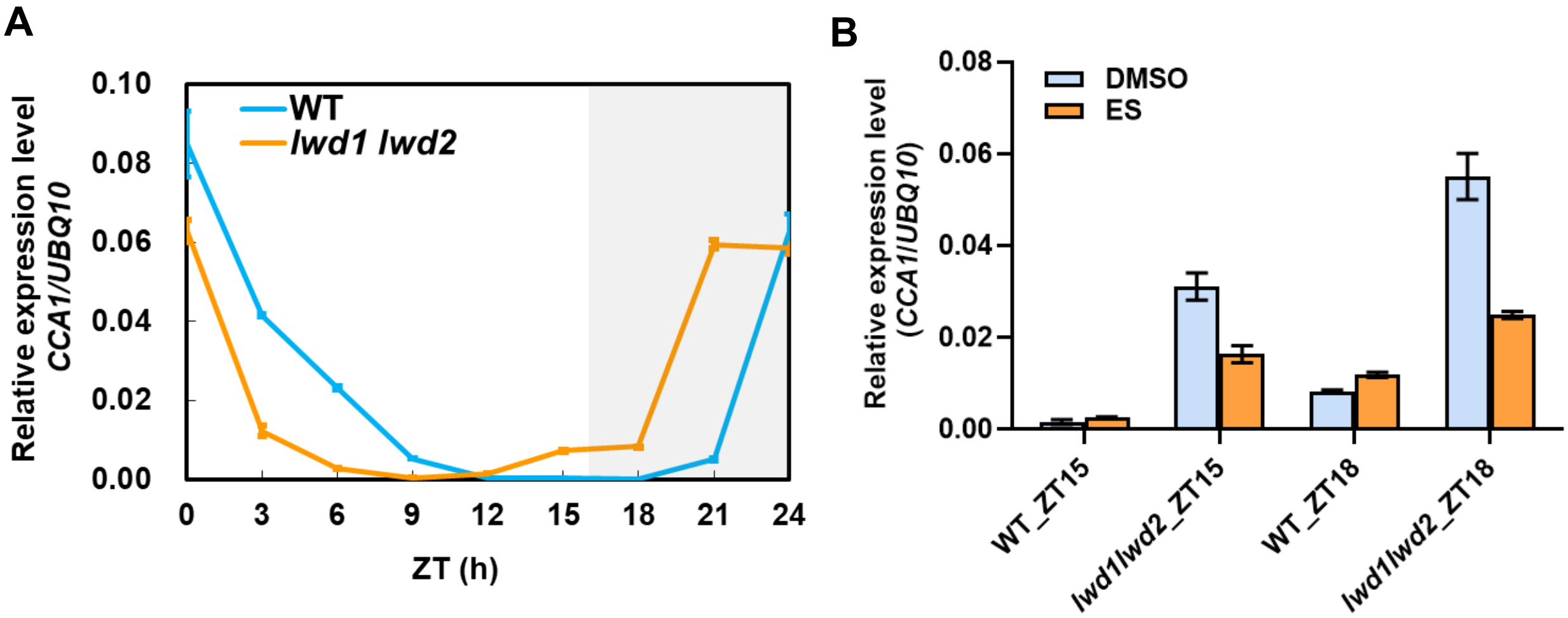

Fig. S14. The expression patterns of *CCA1* upon the induction of *LWD1-HA-YFP* in the *lwd1 lwd2* mutant. **(A)** Diurnal expression of *CCA1* in WT and *lwd1 lwd2* double mutants. Seedlings were grown under long-day conditions (16-h light/8-h dark) for 16 days. Samples were harvested at 3-hr intervals as indicated by Zeitgeber Time (ZT). White and shaded areas represent the light and dark periods, respectively. Relative expression levels of *CCA1* were normalized to *UBQ10* by qRT-PCR. Data are means with standard errors (s.e.) from technical replicates. Three independent biological replicates showed similar results. **(B)** Repression of *CCA1* expression following the induction of *LWD1-HA-YFP* by estradiol (ES). Total RNA were extracted at ZT15 and ZT18 with DMSO (control) or ES treated at ZT3 in the WT and *lwd1 lwd2 pER:LWD1-HA-YFP* seedlings. Relative expression levels of *CCA1* were normalized to *UBQ10.* Error bars represent standard errors (s.e.) of three biological replicates.

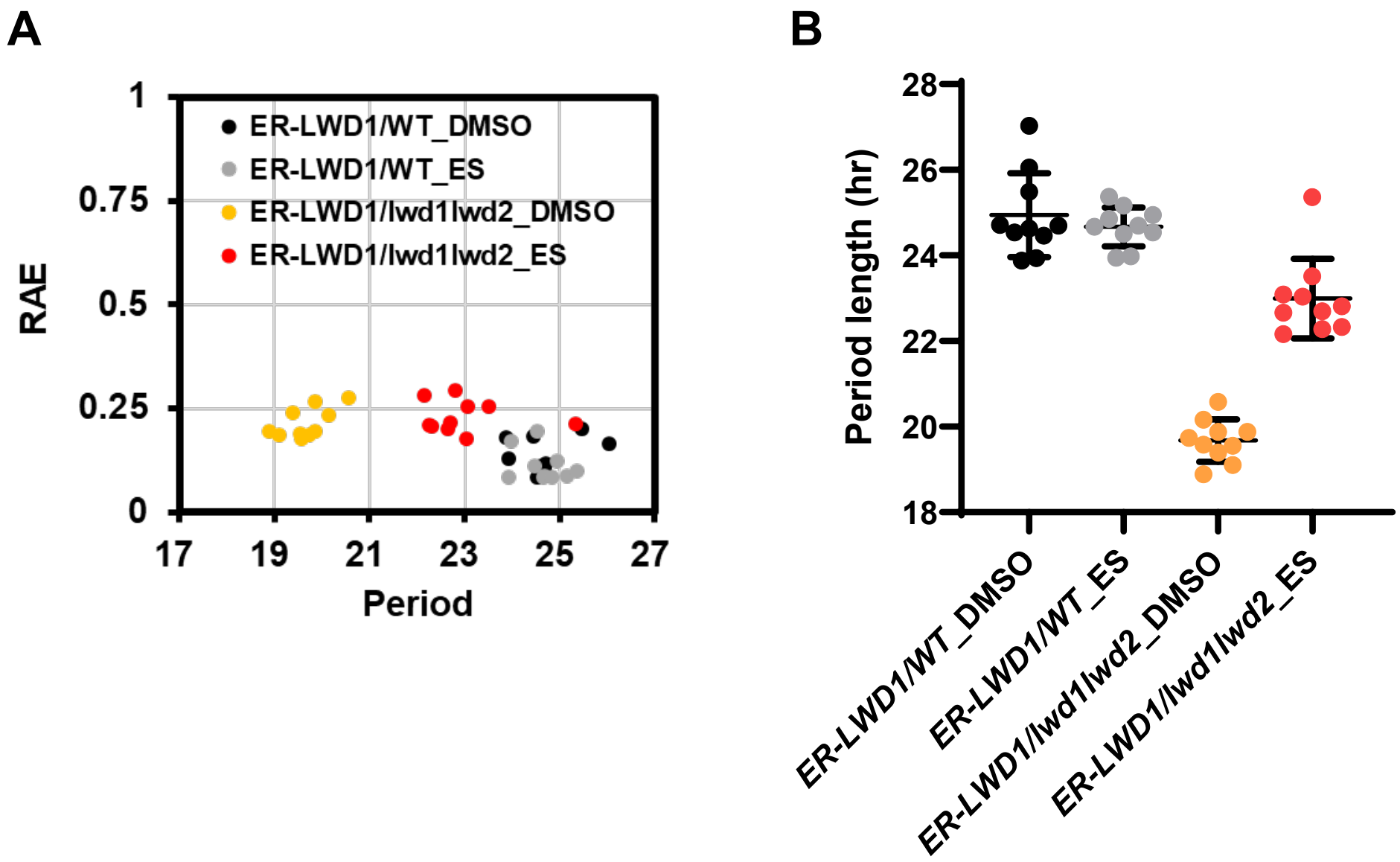

Fig. S15. The induced expression of *LWD1-HA-EYFP* restores the short circadian period phenotype in *lwd1lwd2* mutant.

(A) The period length and relative amplitude error (RAE) were determined by FFT-NLLS analysis with 95% confidence by using the LL80-LL152 in Fig. 6A. **(B)** A bar graph showing the circadian period lengths calculated. Data are means with standard deviations (s.d.) of ≥ 10 seedlings. Three independent experiments were performed with similar results.

References

1. J. F. Wu, Y. Wang, S. H. Wu, Two new clock proteins, LWD1 and LWD2, regulate Arabidopsis photoperiodic flowering. *Plant Physiol* **148**, 948-959 (2008).
2. Y. Wang *et al.*, LIGHT-REGULATED WD1 and PSEUDO-RESPONSE REGULATOR9 form a positive feedback regulatory loop in the Arabidopsis circadian clock. *Plant Cell* **23**, 486-498 (2011).
3. K. A. Kaczorowski, *Mutants in Phytochrome-dependent Seedling Photomorphogenesis and Control of the Arabidopsis Circadian Clock* (University of California, Berkeley, 2004).
4. Y. Yamamoto *et al.*, Comparative genetic studies on the APRR5 and APRR7 genes belonging to the APRR1/TOC1 quintet implicated in circadian rhythm, control of flowering time, and early photomorphogenesis. *Plant Cell Physiol* **44**, 1119-1130 (2003).
5. S. Ito *et al.*, Characterization of the APRR9 pseudo-response regulator belonging to the APRR1/TOC1 quintet in Arabidopsis thaliana. *Plant Cell Physiol* **44**, 1237-1245 (2003).
6. T. C. Branon *et al.*, Efficient proximity labeling in living cells and organisms with TurboID. *Nat Biotechnol* **36**, 880-887 (2018).
7. S. J. Clough, A. F. Bent, Floral dip: a simplified method for Agrobacterium-mediated transformation of Arabidopsis thaliana. *Plant J* **16**, 735-743 (1998).
8. C. S. Chang, J. N. Maloof, S. H. Wu, COP1-mediated degradation of BBX22/LZF1 optimizes seedling development in Arabidopsis. *Plant Physiol* **156**, 228-239 (2011).
9. J. F. Wu *et al.*, LWD-TCP complex activates the morning gene CCA1 in Arabidopsis. *Nat Commun* **7**, 13181 (2016).
10. S. D. Yoo, Y. H. Cho, J. Sheen, Arabidopsis mesophyll protoplasts: a versatile cell system for transient gene expression analysis. *Nat Protoc* **2**, 1565-1572 (2007).
11. F. H. Wu *et al.*, Tape-Arabidopsis Sandwich - a simpler Arabidopsis protoplast isolation method. *Plant Methods* **5**, 16 (2009).
12. C. W. Chen, C. F. Tsai, M. H. Lin, S. Y. Lin, C. C. Hsu, Suspension Trapping-Based Sample Preparation Workflow for In-Depth Plant Phosphoproteomics. *Anal Chem* **95**, 12232-12239 (2023).
13. J. Willforss, A. Chawade, F. Levander, NormalyzerDE: Online Tool for Improved Normalization of Omics Expression Data and High-Sensitivity Differential Expression Analysis. *J Proteome Res* **18**, 732-740 (2019).

14. G. K. Smyth, Linear models and empirical bayes methods for assessing differential expression in microarray experiments. *Stat Appl Genet Mol Biol* **3**, Article3 (2004).
15. W. D. Lin, Y. C. Chen, J. M. Ho, C. D. Hsiao, GOBU: Toward an integration interface for biological objects. *J Inf Sci Eng* **22**, 19-29 (2006).
16. D. Szklarczyk *et al.*, The STRING database in 2021: customizable protein-protein networks, and functional characterization of user-uploaded gene/measurement sets. *Nucleic Acids Res* **49**, D605-D612 (2021).
17. P. Shannon *et al.*, Cytoscape: a software environment for integrated models of biomolecular interaction networks. *Genome Res* **13**, 2498-2504 (2003).
18. V. Citovsky *et al.*, Subcellular localization of interacting proteins by bimolecular fluorescence complementation in planta. *J Mol Biol* **362**, 1120-1131 (2006).
19. D. Yu, G. Chojnowski, M. Rosenthal, J. Kosinski, AlphaPulldown-a python package for protein-protein interaction screens using AlphaFold-Multimer. *Bioinformatics* **39** (2023).
20. M. Mirdita *et al.*, ColabFold: making protein folding accessible to all. *Nat Methods* **19**, 679-682 (2022).
21. J. D. Plautz *et al.*, Quantitative analysis of Drosophila period gene transcription in living animals. *J Biol Rhythms* **12**, 204-217 (1997).
22. M. M. Southern, P. E. Brown, A. Hall, Luciferases as reporter genes. *Methods Mol Biol* **323**, 293-305 (2006).
23. J. Schindelin *et al.*, Fiji: an open-source platform for biological-image analysis. *Nat Methods* **9**, 676-682 (2012).
24. R. D. Gietz, R. H. Schiestl, High-efficiency yeast transformation using the LiAc/SS carrier DNA/PEG method. *Nat Protoc* **2**, 31-34 (2007).
25. S. Chang, J. Puryear, J. Cairney, A simple and efficient method for isolating RNA from pine trees. *Plant Molecular Biology Reporter* **11**, 113-116 (1993).
26. S. L. Yang, N. Tran, M. Y. Tsai, C. K. Ho, Misregulation of MYB16 expression causes stomatal cluster formation by disrupting polarity during asymmetric cell divisions. *Plant Cell* **34**, 455-476 (2022).
27. Y. C. Lee, P. T. Tsai, X. X. Huang, H. L. Tsai, Family Members Additively Repress the Ectopic Expression of BASIC PENTACYSTEINE3 to Prevent Disorders in Arabidopsis Circadian Vegetative Development. *Front Plant Sci* **13**, 919946 (2022).
28. A. V. Gendrel, Z. Lippman, R. Martienssen, V. Colot, Profiling histone modification patterns in plants using genomic tiling microarrays. *Nat Methods* **2**, 213-218 (2005).
